# Research trends and potential molecular intersections between diabetic kidney disease and sarcopenia: a 21-year bibliometric and bioinformatics analysis

**DOI:** 10.3389/fendo.2026.1798210

**Published:** 2026-06-09

**Authors:** Fengling Liu, Hanyu Liu, Sihan Peng

**Affiliations:** 1 Chengdu University of Traditional Chinese Medicine, Chengdu, China; 2 Hospital of Chengdu University of Traditional Chinese Medicine, Chengdu, China; 3 Traditional Chinese Medicine (TCM) Regulating Metabolic Diseases Key Laboratory of Sichuan Province, Hospital of Chengdu University of Traditional Chinese Medicine, Chengdu, China

**Keywords:** diabetic kidney disease, sarcopenia, bibliometric analysis, bioinformatics, molecular overlap, comorbidity

## Abstract

**Background:**

Diabetic kidney disease (DKD) and sarcopenia are increasingly recognized as clinically relevant and potentially interrelated conditions in diabetes, aging, metabolic dysfunction, and functional decline. However, the global research landscape, evolving hotspots, and potential molecular overlap between DKD and sarcopenia remain insufficiently characterized.

**Methods:**

Publications on DKD and sarcopenia from 2005 to 2025 were retrieved from the Web of Science Core Collection, Scopus, and PubMed. After data cleaning, document-type screening, and deduplication, bibliometric analyses were performed using R, VOSviewer, and CiteSpace to assess publication trends, collaboration networks, keyword co-occurrence, thematic evolution, and burst keywords. For exploratory and hypothesis-generating bioinformatics analysis, DKD- and sarcopenia-associated genes were retrieved from GeneCards based on relevance score thresholds defined at the tenths place (DKD ≥ 39.4; sarcopenia ≥ 63.0). Shared genes were identified by Venn analysis and further examined using STRING-based protein–protein interaction analysis, Cytoscape/CytoHubba topological screening, and Gene Ontology and KEGG enrichment analyses with clusterProfiler.

**Results:**

DKD–sarcopenia research showed an overall increasing publication trend over the past two decades. Japan, China, the United States, Italy, and the United Kingdom were major contributors, and several Asian institutions showed prominent productivity. Keyword analyses indicated that hotspots mainly involved diabetes mellitus, sarcopenia, muscle strength, renal dysfunction, hemodialysis, inflammation, insulin resistance, physical performance, and aging-related metabolic disorders. Burst keyword and timeline analyses suggested a gradual shift from descriptive clinical and renal dysfunction-related topics toward functional assessment, comorbidity patterns, dialysis populations, and systemic metabolic complications. In the exploratory and hypothesis-generating gene overlap analysis, 761 overlapping candidate genes were identified between sarcopenia and DKD. These genes were mainly primarily found to be associated with oxygen and hypoxia response, energy metabolism, peptide hormone signaling, protein phosphorylation regulation, growth factor activity, insulin receptor binding, PI3K-Akt signaling, MAPK signaling, AGE-RAGE signaling in diabetic complications, FoxO signaling, HIF-1 signaling, diabetic cardiomyopathy, and cellular senescence.

**Conclusion:**

This study provides an updated bibliometric overview of DKD–sarcopenia research and identifies potential molecular intersections between the two conditions. The findings suggest that inflammation, metabolic dysregulation, hypoxia response, insulin/growth-factor signaling, and cellular stress may represent important directions for future investigation. However, the molecular findings are exploratory and hypothesis-generating rather than direct mechanistic evidence.

## 1 Introduction

With the acceleration of global population aging, the health burden caused by the coexistence of chronic metabolic diseases and geriatric syndromes has become increasingly prominent. Diabetic kidney disease (DKD) is one of the most serious microvascular complications of diabetes, characterized mainly by glomerulosclerosis, tubulointerstitial fibrosis, and progressive decline in renal function. With the continuously increasing global prevalence of diabetes, DKD has become a major contributor to end-stage kidney disease (ESKD), cardiovascular events, premature mortality, and rising healthcare costs, accounting for approximately 50% of ESKD cases worldwide (1, 2). Sarcopenia is a geriatric syndrome characterized by progressive loss of skeletal muscle mass, muscle strength, and physical performance. It is relatively common among patients with diabetes, particularly in older adults (3, 4).

The coexistence of DKD and sarcopenia cannot be fully explained by shared risk factors such as aging alone. Increasing evidence suggests that there may be mutually reinforcing pathophysiological links between these two conditions. In patients with DKD or advanced chronic kidney disease, uremic toxin accumulation, chronic low-grade inflammation, oxidative stress, insulin resistance, metabolic acidosis, protein-energy wasting, reduced physical activity, and dialysis-related amino acid loss may all promote skeletal muscle protein degradation, inhibit muscle synthesis and repair, and thereby accelerate the decline in muscle mass and strength. Conversely, sarcopenia may further impair glycemic control and renal prognosis by reducing skeletal muscle glucose uptake and utilization, aggravating insulin resistance, limiting physical activity, and increasing the risk of frailty. Therefore, DKD and sarcopenia may not represent two independent complications, but rather a “kidney–muscle” comorbid phenotype characterized by metabolic dysregulation, inflammation, and functional decline. Previous studies have shown that the prevalence of severe sarcopenia is markedly increased among dialysis patients and is associated with a higher risk of mortality (5, 6). In addition, a cohort study found that patients with type 2 diabetes and sarcopenia had a higher risk of developing severe DKD than those without sarcopenia (7). These findings highlight the importance of systematically summarizing the research progress and potential intersecting mechanisms between DKD and sarcopenia.

Although the relationship between DKD and sarcopenia has attracted increasing attention, several gaps remain in the current literature. First, the global research landscape of this field has not yet been systematically mapped, including annual publication trends, major contributing countries, institutions, authors, journals, and collaboration networks. Second, the research hotspots and their temporal evolution remain unclear, making it difficult to determine how this field has gradually shifted from early clinical description to mechanistic exploration, risk assessment, and intervention research. Third, although inflammation, insulin resistance, oxidative stress, mitochondrial dysfunction, and metabolic disorders have been implicated in both DKD and sarcopenia, the potential molecular intersections between these two conditions remain insufficiently integrated. Previous studies have mainly focused on specific populations, single mechanisms, or local pathways, which is not sufficient to comprehensively characterize the DKD–sarcopenia comorbidity relationship from both macro-level research trends and micro-level molecular networks.

Bibliometric analysis can reveal research activity, collaboration structures, and thematic evolution within a given field, but it cannot by itself establish biological causality (8, 9). Similarly, disease-related target screening, protein–protein interaction networks, and functional enrichment analyses can be used to identify potential shared genes and pathways, but these results should be regarded as exploratory and hypothesis-generating findings rather than validated pathogenic mechanisms or therapeutic targets. Therefore, integrating bibliometric mapping with molecular network analysis may help describe the developmental trajectory of DKD and sarcopenia research from a descriptive perspective and provide candidate directions for subsequent experimental studies and clinical validation.

Based on this rationale, the present study conducted a bibliometric analysis and exploratory, hypothesis-generating molecular network analysis of DKD- and sarcopenia-related literature from 2005 to 2025, providing a 21-year perspective on this field. At the macro level, we analyzed annual publication trends, contributions of countries and institutions, distributions of authors and journals, collaboration networks, keyword co-occurrence, thematic evolution, and citation characteristics. At the micro level, we integrated disease-related targets, constructed a protein–protein interaction network, and performed functional enrichment analysis to explore the biological processes and signaling pathways that may be shared between DKD and sarcopenia. This study aims to systematically depict the research landscape of the DKD–sarcopenia field, identify major research hotspots and potential frontier directions, and propose hypotheses for further experimental and clinical validation, emphasizing that our bioinformatics results reflect database-level associations rather than causal mechanisms or validated therapeutic targets.

## 2 Materials and methods

### 2.1 Data sources and search strategy

This study adopted a combined multi-database search strategy to systematically retrieve literature related to DKD and sarcopenia or muscle loss from three databases: Web of Science Core Collection (WoSCC), Scopus, and PubMed. These three databases were selected for the following reasons: WoSCC and Scopus have advantages in interdisciplinary literature coverage, citation indexing, and reference fields, making them suitable for bibliometric and knowledge structure analyses; PubMed, as an important database in the biomedical field, can supplement literature related to clinical and basic medical research, thereby improving retrieval coverage and reducing potential bias caused by reliance on a single database (10–12).

The search strategy was constructed using a combination of a “disease module” and a “muscle loss module.” The disease module mainly included terms such as “diabetic nephropathy,” “diabetic kidney disease,” “diabetic renal disease,” “diabetes-related kidney disease,” “diabetes-associated kidney disease,” “chronic diabetic nephropathy,” “kidney failure in diabetes,” “renal dysfunction in diabetes,” “diabetic kidney dysfunction,” “diabetic renal impairment,” and “nephropathy in diabetes.” The muscle loss module mainly included terms such as “sarcopenia,” “cachexia,” “muscle wasting,” “muscle atrophy,” “muscle mass loss,” “muscle depletion,” “skeletal muscle wasting,” “skeletal muscle dysfunction,” and “muscle weakness.” All records were required to meet both the DKD-related module and the muscle loss-related module to improve topic relevance and reduce thematic contamination caused by separately searching chronic kidney disease, diabetes, or sarcopenia. Field tags, Boolean operators, and proximity operators were adjusted according to the search syntax of each database. The complete search strategies are provided in **Supplementary Table 1**. The search time span was set from database inception to December 31, 2025. Considering that publications in 2026 were still undergoing continuous publication, indexing, and dynamic supplementation, their direct inclusion might introduce time-truncation bias and affect the stability of analyses of annual publication trends, collaboration structures, and thematic evolution. Therefore, records from 2026 were excluded from the formal analysis. The document types were limited to Articles and Reviews, and the language was restricted to English (13, 14).

### 2.2 Literature export, data integration, and screening process

After completing the searches, bibliographic records were exported separately from the three databases. WoSCC records were exported in full-record plain-text format, Scopus records were exported in CSV format, and PubMed records were exported in Citation Manager (.nbib) format. All records were then imported into the R environment and converted into a unified bibliographic data structure using the bibliometrix package to support cross-database integration analysis.

A total of 1,323 records were initially retrieved, including 198 from WoSCC, 509 from Scopus, and 616 from PubMed. Records published or indexed in 2026 were first excluded, together with records with missing year information or records for which the publication year could not be clearly determined, leaving 1,169 records. The document types were then uniformly screened, retaining only Articles and Reviews and excluding conference papers, meeting abstracts, editorials, letters, news items, corrections, book chapters, and other non-research records. After this step, 1,161 records remained.

**Figure 1:**
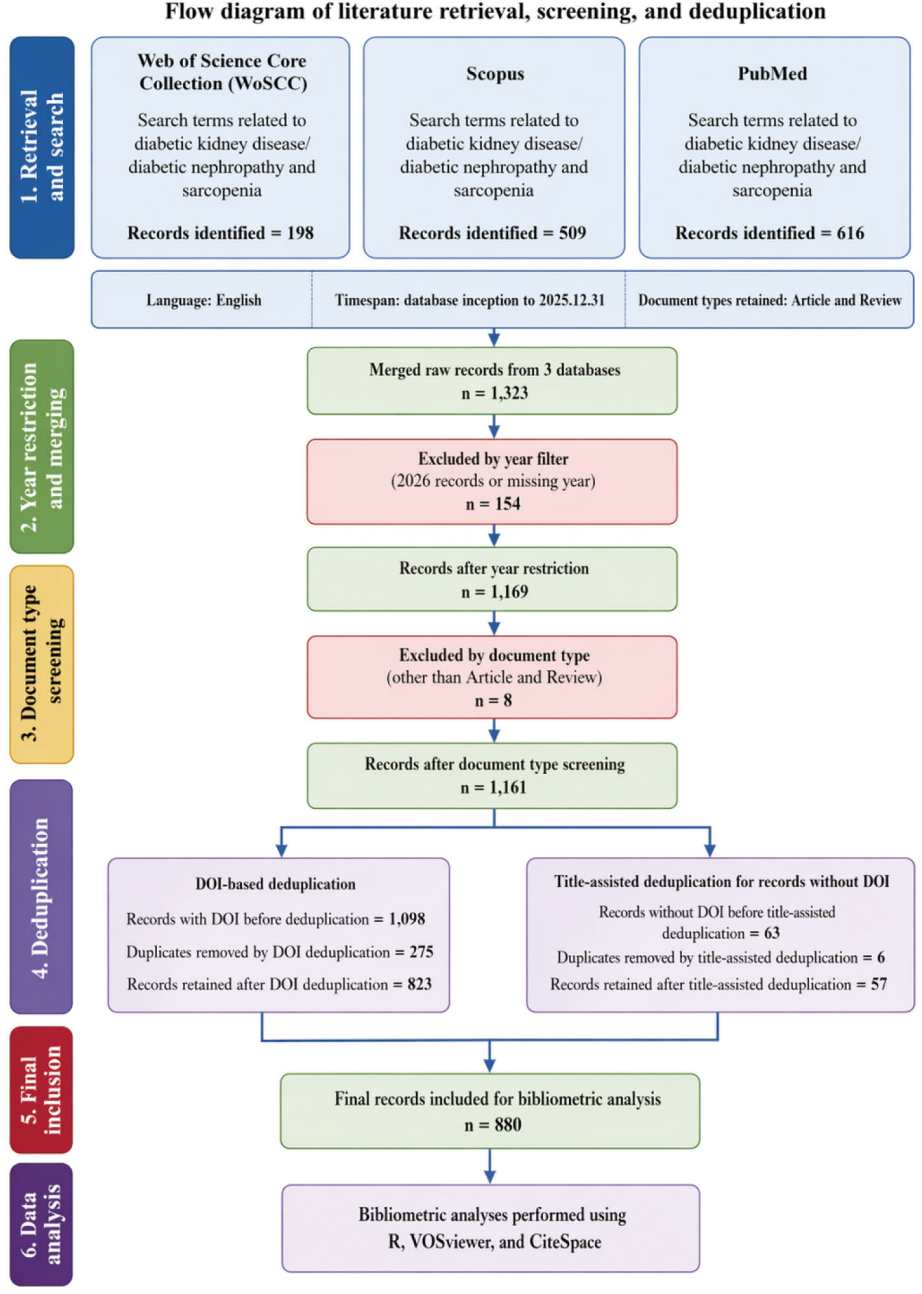
Literature screening and data processing workflow.

Duplicate records were identified using a two-step strategy of “DOI first, title-assisted deduplication.” First, DOI fields were standardized by unifying letter case, removing prefixes such as “https://doi.org/” and “doi:”, and eliminating extra spaces and other formatting inconsistencies. Among the 1,098 records with DOI information, exact matching based on standardized DOI was performed, and 823 records were retained after deduplication. For the 63 records without DOI information, title-assisted deduplication was performed. Titles were first standardized by normalizing letter case, punctuation, and spaces, and duplicates were then identified in combination with first author and publication year information. Finally, 57 records without DOI information were retained. After the above screening and deduplication procedures, a standardized dataset of 880 publications was obtained for the main bibliometric analyses. The complete screening and deduplication process is shown in **Figure 1**.

### 2.3 Data cleaning and standardization

To improve the accuracy of cross-database integration, this study standardized the fields of authors, countries/regions, institutions, journals, and keywords. For country/region names, obvious synonyms or variant expressions were merged. For example, “UNITED STATES” and “United States” were merged into “United States”; “CHINA,” “PEOPLES R CHINA,” and “CN” were merged into “China”; and “SOUTH KOREA,” “REPUBLIC OF KOREA,” and “KOREA” were merged into “South Korea.” In addition, non-country labels generated by email addresses, institutional addresses, or field parsing errors, such as “COM,” “EDU,” and “AC,” were removed.

For institutional names, obvious abbreviations, case differences, and name variants among high-frequency institutions were merged. For journal names, full names and obvious formatting variants were standardized to reduce duplicate counting. For keywords, letter case was first unified, and redundant symbols and spaces were removed. Synonymous or closely related expressions were then merged where appropriate. For example, “diabetic nephropathy,” “diabetic kidney disease,” and “DKD” were assigned to a relatively consistent conceptual framework of DKD; similarly, muscle loss-related terms such as “sarcopenia,” “muscle wasting,” “muscle atrophy,” and “muscle weakness” were moderately standardized. The cleaned dataset was used for the main analyses, while the raw data and selected cleaning results are available from the corresponding author upon request to enhance the traceability of the study.

### 2.4 Bibliometric analysis

Except for citation relationship analyses, the main bibliometric analyses in this study were based on the final merged dataset from WoSCC, Scopus, and PubMed. The analyses included annual publication trends, country/region distribution, international collaboration patterns, institutional output and collaboration networks, author productivity, journal distribution, keyword frequency, keyword co-occurrence networks, and the evolution of research hotspots.

Annual publication trends were used to describe the overall developmental trajectory of DKD- and sarcopenia-related research. Analyses of countries/regions, institutions, authors, and journals were used to identify the major contributors and core publication platforms in this field. Country/region collaboration was evaluated using single-country publications (SCP) and multiple-country publications (MCP). SCP refers to publications in which all author affiliations were from the same country, whereas MCP refers to publications involving author affiliations from two or more countries. MCP% was calculated as MCP divided by the total number of publications multiplied by 100%, reflecting the proportion of international collaboration in the publications of a given country or region.

Annual publication trends were presented using descriptive statistics and trend curves. Trend fitting was used only as a visual aid to describe changes in research activity and was not intended to predict future publication volume. To avoid overfitting, model selection considered goodness of fit, curve stability, and interpretive simplicity. All interpretations of trends were limited to describing temporal changes in research attention and literature output in this field, and were not interpreted as changes in disease mechanisms or causal relationships.

### 2.5 Visualization analysis

VOSviewer was used to visualize country/region collaboration, author collaboration, institutional collaboration, and keyword co-occurrence networks (15). The analysis types included co-authorship and co-occurrence, and the counting method was full counting. Networks of countries/regions, authors, institutions, and keywords were used to display the international collaboration landscape, core author groups, major research institutions, and associations among research hotspot themes.

In the keyword co-occurrence analysis, the unit of analysis was set as all keywords, and full counting was used. To retain the main thematic structure while reducing visual noise caused by low-frequency keywords, a minimum keyword occurrence threshold was set. This threshold was determined after comparing network size, node density, cluster clarity, and graphical readability under different thresholds. The purpose was to highlight stable and interpretable core themes without excessively introducing incidental or marginal terms.

CiteSpace was used for keyword clustering, timeline visualization, and burst term analysis to assist in identifying the evolution of research themes and potential research frontiers. CiteSpace results were mainly used to explore thematic transitions and changes in hotspots, rather than as evidence for causal mechanism inference or primary citation conclusions (15, 16).

### 2.6 Citation performance analysis

Considering that different databases vary in coverage, reference field structure, and citation-counting rules, and that PubMed in particular does not provide citation and reference information with the same level of structural detail as WoSCC and Scopus, this study treated overall bibliometric analysis and citation performance analysis separately. The merged three-database dataset was mainly used to describe research output trends, country/region and institutional collaboration networks, author and journal distributions, and keyword co-occurrence patterns, thereby improving the comprehensiveness of literature coverage. In contrast, citation performance analysis was conducted only on records with available citation fields and reference information, and was used as a descriptive indicator to identify representative high-impact publications, source journals, and countries/regions.

In the citation performance analysis, global citation counts and local citation counts were mainly calculated. Global citation counts were used to reflect the overall influence of publications within the broader academic community, while local citation counts were used to reflect how often a publication was cited by other publications included in the present dataset, thereby indicating its knowledge-base role within the intersecting field of DKD and sarcopenia research. Based on these indicators, globally highly cited publications and locally highly cited publications were summarized separately to identify representative key studies in this field. It should be noted that citation counts are susceptible to publication year, database coverage, journal visibility, and citation lag effects. Therefore, citation results were interpreted descriptively, and high citation counts were not directly equated with evidence quality, mechanistic certainty, or clinical importance. This approach preserved the coverage advantage of multi-database retrieval while reducing interpretive bias caused by heterogeneity in cross-database citation data (17).

### 2.7 Bioinformatics analysis

To preliminarily explore potential biological intersections between DKD and sarcopenia at the molecular level, this study conducted an exploratory bioinformatics analysis. Disease-related gene sets were obtained from the GeneCards database. DKD-related genes were retrieved using the terms “diabetic kidney disease” and “diabetic nephropathy,” while sarcopenia-related genes were retrieved using “sarcopenia” and “muscle atrophy.” Candidate genes were screened according to predefined thresholds based on the GeneCards relevance score(defined at the tenths place: DKD ≥ 39.4 and sarcopenia ≥ 63.0.). The two disease-related gene sets were then imported into the R environment(R version 4.3.1), and shared candidate genes between DKD and sarcopenia were obtained through Venn analysis.The statistical significance of the overlap was assessed using hypergeometric test (p < 0.001).

The shared candidate genes were submitted to the STRING database(version 11.5) to construct a protein–protein interaction (PPI) network. The species was restricted to Homo sapiens, and a minimum interaction confidence score of 0.900 (high confidence) was set to improve network reliability. The PPI network was then imported into Cytoscape (version 3.9.1) for visualization and topological analysis. The CytoHubba plugin was used to calculate network topological parameters, including Degree and Betweenness Centrality, and potential hub genes were identified according to the MCC (Maximal Clique Centrality) algorithm.

Functional enrichment analysis was performed in the R environment. Gene ID conversion was conducted using the org.Hs.eg.db package(version 3.18.0), and Gene Ontology (GO) functional enrichment analysis and Kyoto Encyclopedia of Genes and Genomes (KEGG) pathway enrichment analysis were performed using the clusterProfiler package(version 4.8.0). The statistical significance threshold was set as adjusted P value < 0.05. Enrichment results were visualized using bar plots, bubble plots, and network diagrams.

It should be emphasized that the bioinformatics analysis in this study was not a differential expression analysis based on a unified transcriptomic dataset, but an exploratory disease-gene intersection analysis based on the GeneCards disease-related gene database. Therefore, the identified shared genes only indicate a database-level association with both DKD and sarcopenia. This analysis lacks information on expression directionality, tissue specificity, and has not been experimentally validated. Consequently, these findings cannot support causal inferences or confirm therapeutic target value. This part of the analysis is positioned strictly as hypothesis-generating, aiming to provide candidate genes and pathway clues for subsequent mechanistic studies.

### 2.8 Statistical transparency and reproducibility

First, justification for the absence of inferential statistics. This study employed descriptive statistics because it analyzed the entire population of retrieved literature rather than a sample. Inferential statistics are designed for sample-to-population generalization, which is not applicable when the complete dataset is available. Therefore, confidence intervals and hypothesis tests were not conducted.

Second, robustness considerations. To enhance result robustness, this study implemented multiple measures: (1) combining the WoSCC, Scopus, and PubMed databases to maximize literature coverage; (2) presenting annual publication trends for each database and for the combined dataset to evaluate source consistency; (3) employing multidimensional metrics (publication trends, keyword evolution, topic clustering) to cross-validate research hotspots, preventing bias from single indicators; (4) applying strict statistical thresholds (hypergeometric test P<0.01 for intersections; adjusted P<0.05 for enrichment).

Third, limitations of descriptive/bibliometric approaches. It should be emphasized that descriptive bibliometric approaches have intrinsic limitations: they capture patterns of scholarly attention and research activity rather than direct evidence for disease mechanisms or causality; additionally, results may be affected by external factors such as database coverage, language biases, and indexing policies, which could lead to selection bias. Consequently, the results of this study should be considered as exploratory instruments for hypothesis generation and identification of knowledge gaps, with conclusions to be corroborated by subsequent empirical research.

Fourth, statement on data accessibility and reproducibility workflow. To ensure reproducibility and transparency, this study provides comprehensive documentation of all methodological procedures. Search strategies for each database are supplied in the Supplementary Materials. The Methods section details database sources, search periods, literature type restrictions, data export formats, cross-database merging and deduplication procedures, field standardization rules, software employed (VOSviewer, CiteSpace, R, etc.) with their specific versions, and primary analysis parameters. For the bioinformatics analyses, all R scripts used for Venn analysis, PPI network construction, topological analysis, and functional enrichment analysis are available from the corresponding author upon reasonable request. Original datasets can be obtained from the corresponding author upon reasonable request to facilitate independent verification of the analytical pipeline.

## 3 Results

### 3.1 Literature screening results

As shown in **Figure 1**, a total of 1,323 initial records were retrieved from three databases, including 198 records from WoSCC, 509 from Scopus, and 616 from PubMed. After year-based screening, 1,169 records were retained. After further limiting the document types to Articles and Reviews, 1,161 records remained. Duplicate records were then removed preferentially based on DOI. Among the 1,098 records with DOI information, 823 records were retained after DOI-based deduplication. For the 63 records without DOI information, auxiliary deduplication was further performed based on title, first author, and publication year, resulting in 57 non-DOI records being retained. After the above screening and deduplication procedures, 880 publications were finally included for subsequent bibliometric analysis.

### 3.2 Annual publication trends

**Figure 2:**
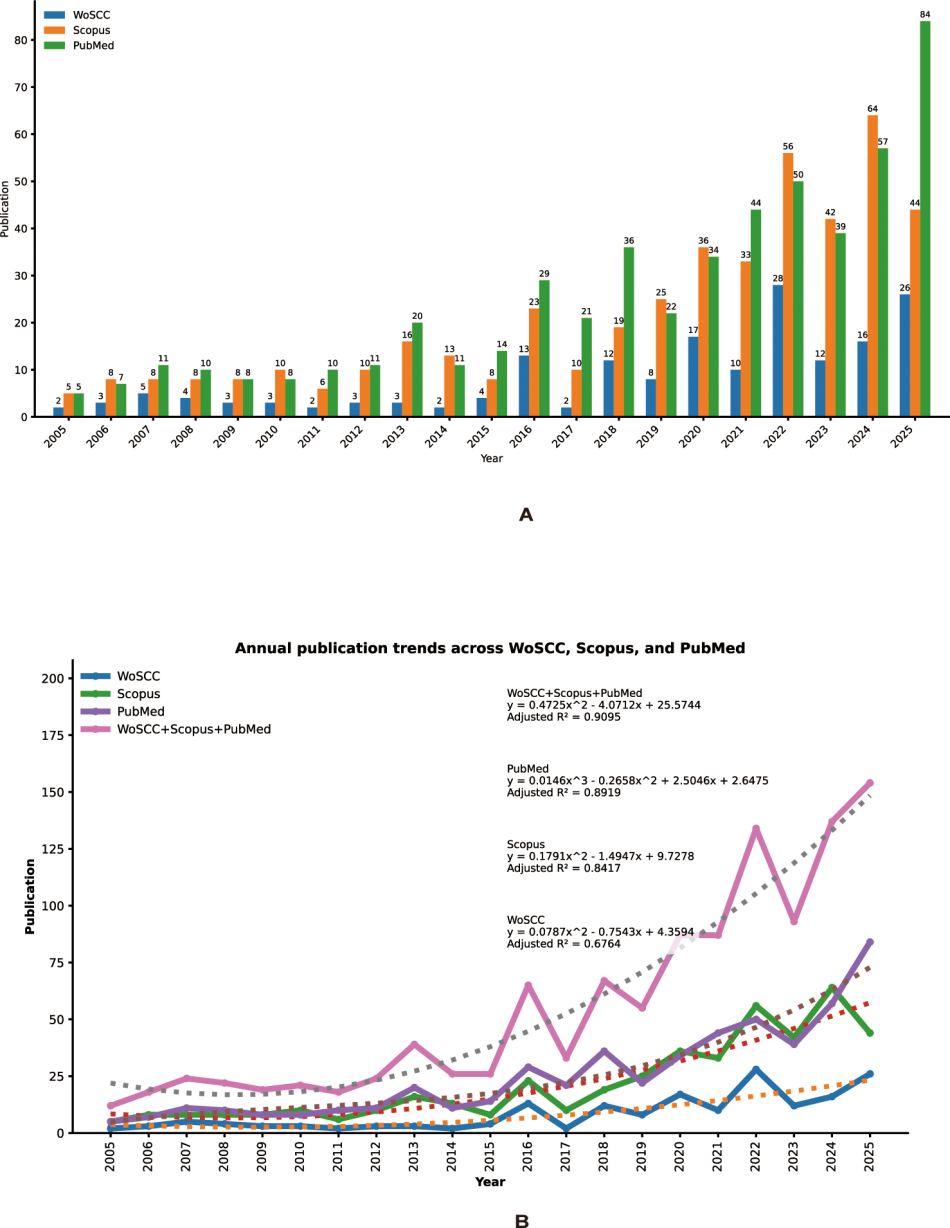
Annual publication trends in diabetic kidney disease and sarcopenia research from 2005 to 2025. **(A)** Annual numbers of publications indexed in Web of Science Core Collection, Scopus, and PubMed. **(B)** Temporal trends of annual publications across individual databases and the combined dataset.

**Figure 2** shows the annual publication trends of studies related to DKD and sarcopenia in the WoSCC, Scopus, and PubMed databases from 2005 to 2025. Overall, the number of publications in this field showed a clear upward trend, although the growth rate varied across different stages. As shown in **Figure 2A**, during 2005–2015, the annual number of publications in each database remained relatively low, suggesting that research in this field was still at an early exploratory stage. After 2016, the number of publications in all three databases began to increase gradually, indicating growing attention to the relationship between diabetic kidney injury and muscle loss, muscle atrophy, and abnormalities in nutritional metabolism. After 2020, annual publication output increased more markedly, especially during 2022–2025, when it reached a relatively high level. This suggests that this topic has recently become a research hotspot at the intersection of diabetic complications, nephrology, geriatrics, and skeletal muscle metabolism.

Across different databases, PubMed contained the highest overall number of publications, with particularly notable growth in recent years, reaching 84 publications in 2025. This reflects the rapidly increasing attention given to this topic in biomedical research. The number of publications in Scopus also showed a continuous upward trend, with relatively high peaks in 2022 and 2024, reaching 56 and 64 publications, respectively. The annual number of publications in WoSCC was relatively lower, but it also showed a gradual increasing trend, reaching 26 publications in 2025. This indicates that research accumulation in this field is also increasing in high-quality citation index databases.

**Figure 2B** further presents the annual publication trends in WoSCC, Scopus, PubMed, and the merged three-database dataset. The overall publication output of the merged dataset gradually shifted from low-level fluctuations in the early period to a stage of rapid growth and reached its highest level in 2025, demonstrating the rapid development of this research direction in recent years. The fitted curves showed a high degree of fit for both the merged dataset and each individual database, indicating a relatively stable growth pattern in research output over time. It should be emphasized that the trend fitting was performed solely for descriptive purposes to visualize temporal changes in research activity, and should not be interpreted as predictive modeling or causal inference regarding disease mechanisms.

Overall, research on DKD and sarcopenia has evolved from scattered early exploration into a continuously expanding interdisciplinary field. The rapid growth observed over the past five years suggests that its importance in clinical risk assessment, nutritional intervention, muscle function preservation, and chronic complication management is increasingly recognized.

### 3.3 Distribution of major source journals and research institutions

**Figure 3:**
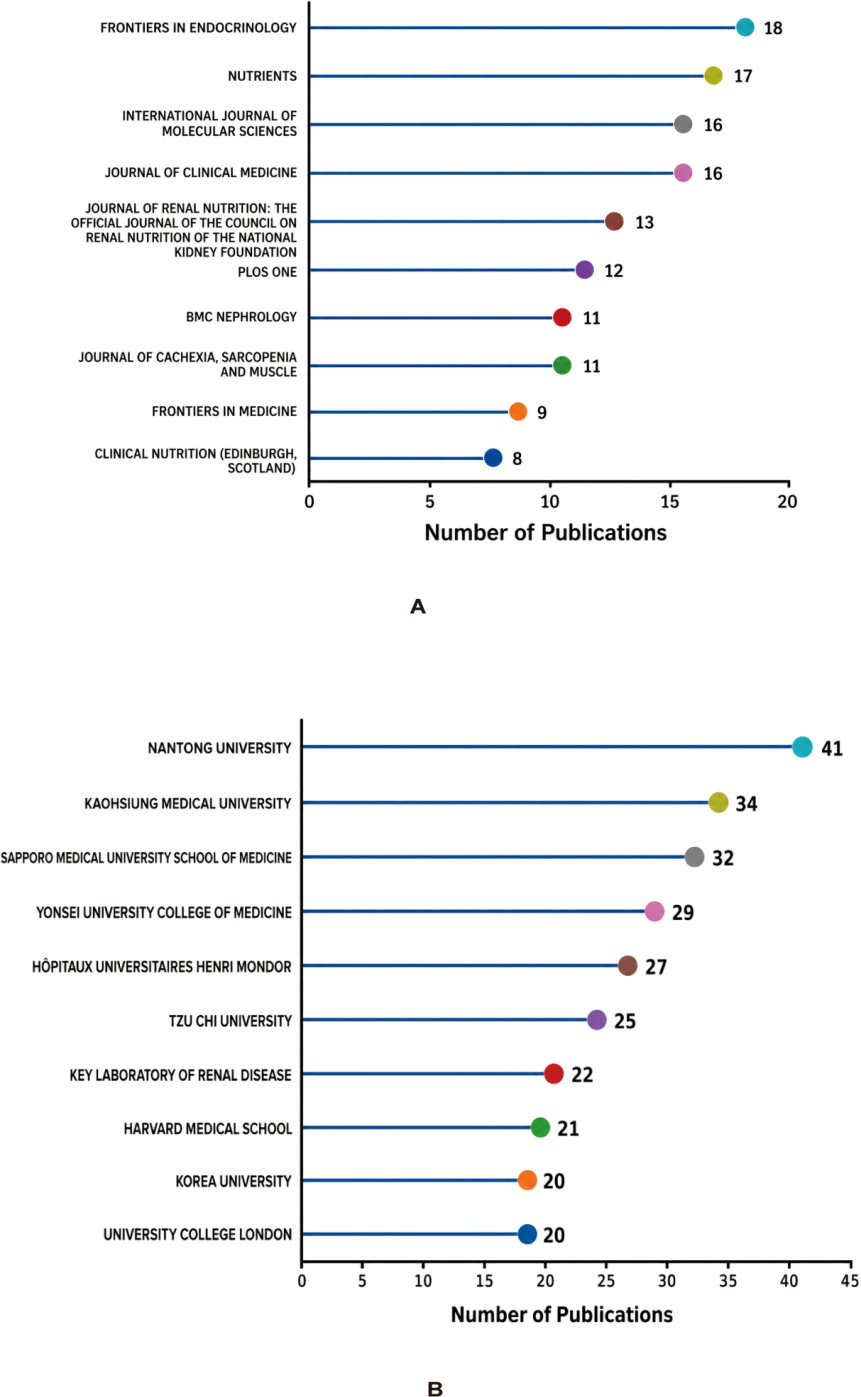
Leading journals, and institutions in diabetic kidney disease and sarcopenia research. **(A)** Top 10 journals ranked by publication output. **(B)** Top 10 institutions ranked by publication.

**Figure 3** shows the top 10 source journals and research institutions in terms of publication output in the field of DKD and sarcopenia. Overall, research output in this field was mainly concentrated in Asia, North America, and Europe, while the source journals were primarily distributed across endocrinology and metabolism, nutrition, nephrology, and sarcopenia-related fields.

As shown in **Figure 3A**, the top 10 source journals were mainly related to endocrinology and metabolism, nutrition, nephrology, and geriatric muscle disorders. Frontiers in Endocrinology ranked first with 18 publications, followed by Nutrients with 17 publications. International Journal of Molecular Sciences and Journal of Clinical Medicine each published 16 articles. Journal of Renal Nutrition published 13 articles, PLOS One published 12 articles, and BMC Nephrology and Journal of Cachexia, Sarcopenia and Muscle each published 11 articles. Frontiers in Medicine and Clinical Nutrition also ranked among the top 10. These results indicate that this field has clear multidisciplinary characteristics, involving diabetic complications, renal nutrition, chronic kidney disease, muscle loss, inflammatory metabolism, and integrated clinical management.

**Figure 3B** shows the top 10 research institutions by publication output. Nantong University ranked first with 41 publications, followed by Kaohsiung Medical University with 34 publications and Sapporo Medical University School of Medicine with 32 publications. Other high-output institutions included Yonsei University College of Medicine with 29 publications, Hôpitaux Universitaires Henri Mondor with 27 publications, Tzu Chi University with 25 publications, Key Laboratory of Renal Disease with 22 publications, Harvard Medical School with 21 publications, Korea University with 20 publications, and University College London with 20 publications. Overall, the high-output institutions were mainly located in China, Japan, South Korea, Taiwan, France, the United States, and the United Kingdom. This suggests that the core research capacity in this field mainly comes from medical universities, affiliated hospitals, and nephrology-related research institutions in Asia, Europe, and North America.

Taken together, research on DKD and sarcopenia shows clear regional concentration and multidisciplinary integration. China, Japan, and the United States are the major publication-producing countries in this field. Core journals mainly cover endocrinology and metabolism, nutrition, nephrology, and sarcopenia research. High-output institutions are mostly medical universities, clinical medical centers, and nephrology research institutions, reflecting the combined clinical and basic mechanistic nature of this field.

### 3.4 Major countries/regions and international collaboration patterns

**Table 1** presents the top 20 countries/regions by publication output and their patterns of single-country and multiple-country publications. Overall, research output in this field was mainly concentrated in Asia, North America, and Europe, with Japan, the United States, and China ranking as the top three contributors to research on DKD and sarcopenia.

**Table 1:** Top 20 countries/regions by publication output after harmonizing duplicate country labels.

Rank	Country/Region	Publications	SCP	MCP	MCP (%)
1	Japan	143	98	45	31.5
2	United States	140	72	68	48.6
3	China	132	92	40	30.3
4	Italy	77	30	47	61.0
5	United Kingdom	71	27	44	62.0
6	South Korea	51	33	18	35.3
7	Taiwan	42	23	19	45.2
8	Brazil	38	18	20	52.6
9	India	35	24	11	31.4
10	Germany	30	9	21	70.0
11	Canada	26	11	15	57.7
12	France	25	13	12	48.0
13	Sweden	25	6	19	76.0
14	Turkey	25	16	9	36.0
15	Australia	22	9	13	59.1
16	Spain	20	6	14	70.0
17	Greece	16	6	10	62.5
18	Poland	14	10	4	28.6
19	Austria	12	4	8	66.7
20	Switzerland	12	3	9	75.0

SCP, single-country publications; MCP, multiple-country publications. MCP (%) was calculated as MCP divided by total publications. Duplicate country labels were harmonized before ranking; non-country fields such as COM, EDU, AC, P, and ST were excluded.

Japan had the highest publication output, with 143 publications, including 98 single-country publications and 45 multiple-country publications, corresponding to an MCP proportion of 31.5%. The United States ranked second with 140 publications, including 68 multiple-country publications and an MCP proportion of 48.6%, indicating a strong international collaboration pattern. China ranked third with 132 publications, including 92 single-country publications and 40 multiple-country publications, with an MCP proportion of 30.3%. This suggests that China has high research productivity in this field, although its level of international collaboration is relatively lower than that of some European and North American countries.

Among European countries, Italy, the United Kingdom, Germany, Sweden, Spain, Austria, and Switzerland had lower total publication output than Japan, the United States, and China, but their proportions of multiple-country publications were relatively high. Sweden had the highest MCP proportion at 76.0%, followed by Switzerland at 75.0%, Germany and Spain both at 70.0%, Austria at 66.7%, the United Kingdom at 62.0%, and Italy at 61.0%. These findings indicate that European countries tend to engage more actively in transnational collaborative research in this field.

By contrast, India, Poland, and China had relatively lower MCP proportions of 31.4%, 28.6%, and 30.3%, respectively, indicating that their research was more often conducted independently by domestic teams. South Korea, Taiwan, Brazil, Canada, France, Turkey, Australia, and Greece showed moderate levels of collaboration, with both a certain degree of domestic research capacity and varying levels of participation in international collaboration.

Overall, **Table 1** indicates that research on DKD and sarcopenia has formed a clear pattern of global participation. Japan, the United States, and China are the leading publication-producing countries in this field. Although the total publication output of European countries is relatively dispersed, their international collaboration activity is high. Asian countries make substantial contributions in terms of publication volume, but some still have room to strengthen transnational collaboration. These findings suggest that future cross-regional cooperation among Asia, Europe, and North America may help promote further development in clinical evidence integration, mechanistic research, and intervention strategy development.

### 3.5 Keyword co-occurrence, clustering, and research frontier analysis

**Figure 4:**
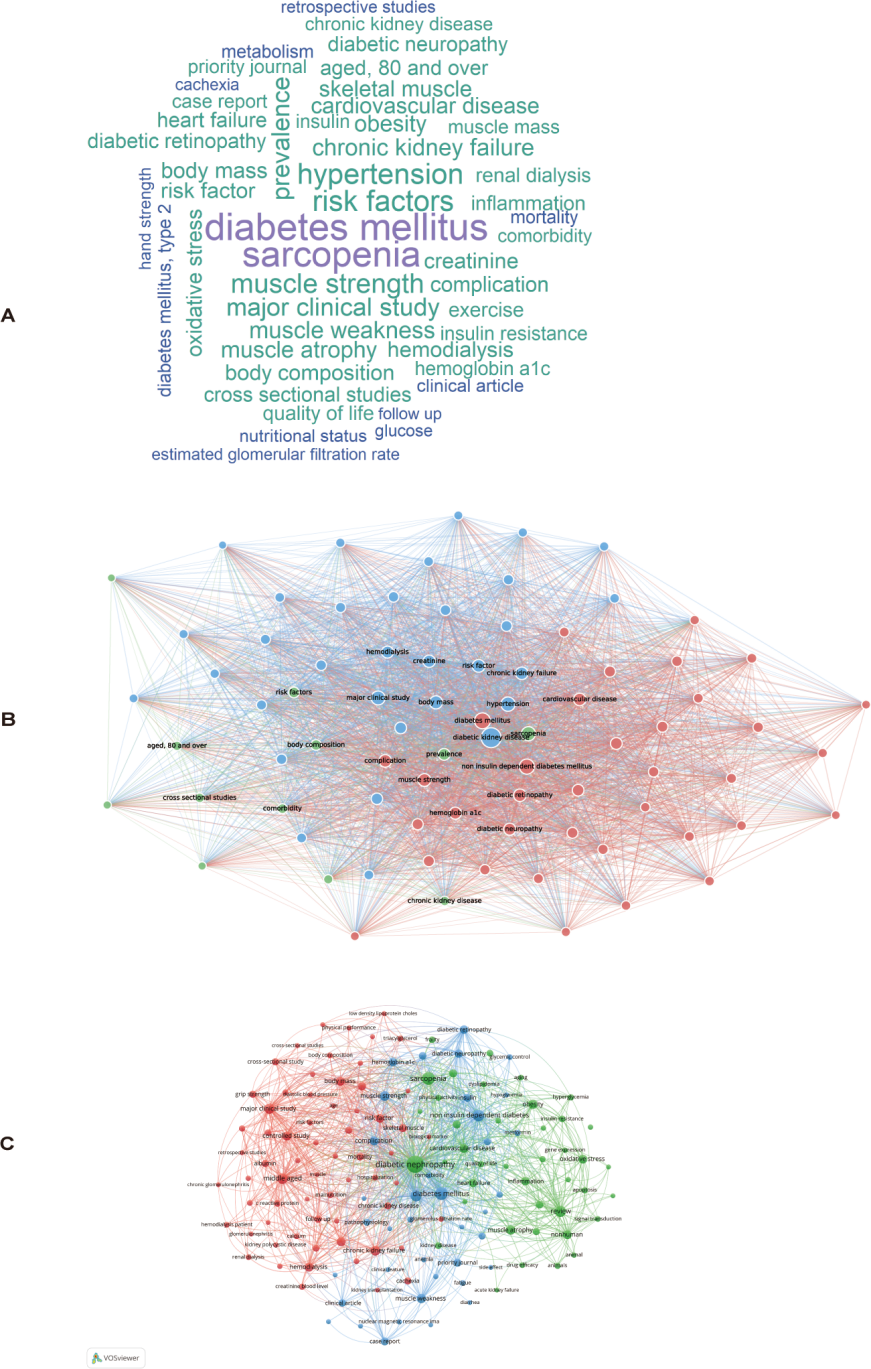
Keyword frequency and co-occurrence structure in diabetic kidney disease and sarcopenia research. **(A)** Word cloud of high-frequency keywords generated in (R) Larger terms indicate higher occurrence frequency. **(B)** Keyword co-occurrence network generated in (R) **(C)** Keyword co-occurrence clustering map generated by VOSviewer.

To reveal the core themes and evolutionary trends in research on DKD and sarcopenia, this study conducted a comprehensive keyword analysis of the included publications. First, high-frequency keyword word clouds and keyword co-occurrence networks were generated using R (**Figures 4A, B**). The word cloud showed that keywords such as diabetes mellitus, sarcopenia, muscle strength, chronic kidney failure, risk factors, hypertension, skeletal muscle, renal dialysis, hemodialysis, muscle mass, muscle weakness, muscle atrophy, body composition, cardiovascular disease, obesity, and insulin resistance appeared frequently. These findings suggest that research in this field mainly focuses on diabetes, renal impairment, decline in muscle mass and strength, dialysis treatment, metabolic abnormalities, and cardiovascular comorbidities. Among these terms, diabetes mellitus and sarcopenia were located at the center of the word cloud, indicating that these two conditions constitute the core disease combination in this research field. Keywords such as muscle strength, muscle mass, and body composition further suggest that the research focus has gradually expanded from disease diagnosis itself to functional status and body composition assessment.

The keyword co-occurrence network constructed using R further showed close connections among high-frequency keywords, forming an association structure centered on diabetes mellitus, sarcopenia, chronic kidney disease/chronic kidney failure, muscle strength, hypertension, creatinine, hemodialysis, and cardiovascular disease. This suggests that research on DKD and sarcopenia is not limited to a single disease outcome, but is closely related to multidimensional factors such as renal function indicators, dialysis status, metabolic abnormalities, cardiovascular risk, and decline in muscle function.

Subsequently, VOSviewer was used to conduct keyword co-occurrence clustering analysis (**Figure 4C**). The results showed that the keyword network broadly formed several interrelated thematic modules. One module was represented by diabetes mellitus, DKD, insulin resistance, obesity, and metabolic syndrome, reflecting themes related to diabetes and metabolic abnormalities. Another module was represented by chronic kidney disease, renal dialysis, hemodialysis, kidney failure, and estimated glomerular filtration rate, reflecting themes related to renal impairment and dialysis treatment. A further module was represented by sarcopenia, skeletal muscle, muscle strength, muscle mass, physical performance, and body composition, reflecting themes related to muscle loss, body composition, and functional assessment. The numerous connections among these modules indicate clear thematic intersections among DKD, renal function decline, sarcopenia phenotypes, and metabolic comorbidities.

**Figure 5:**
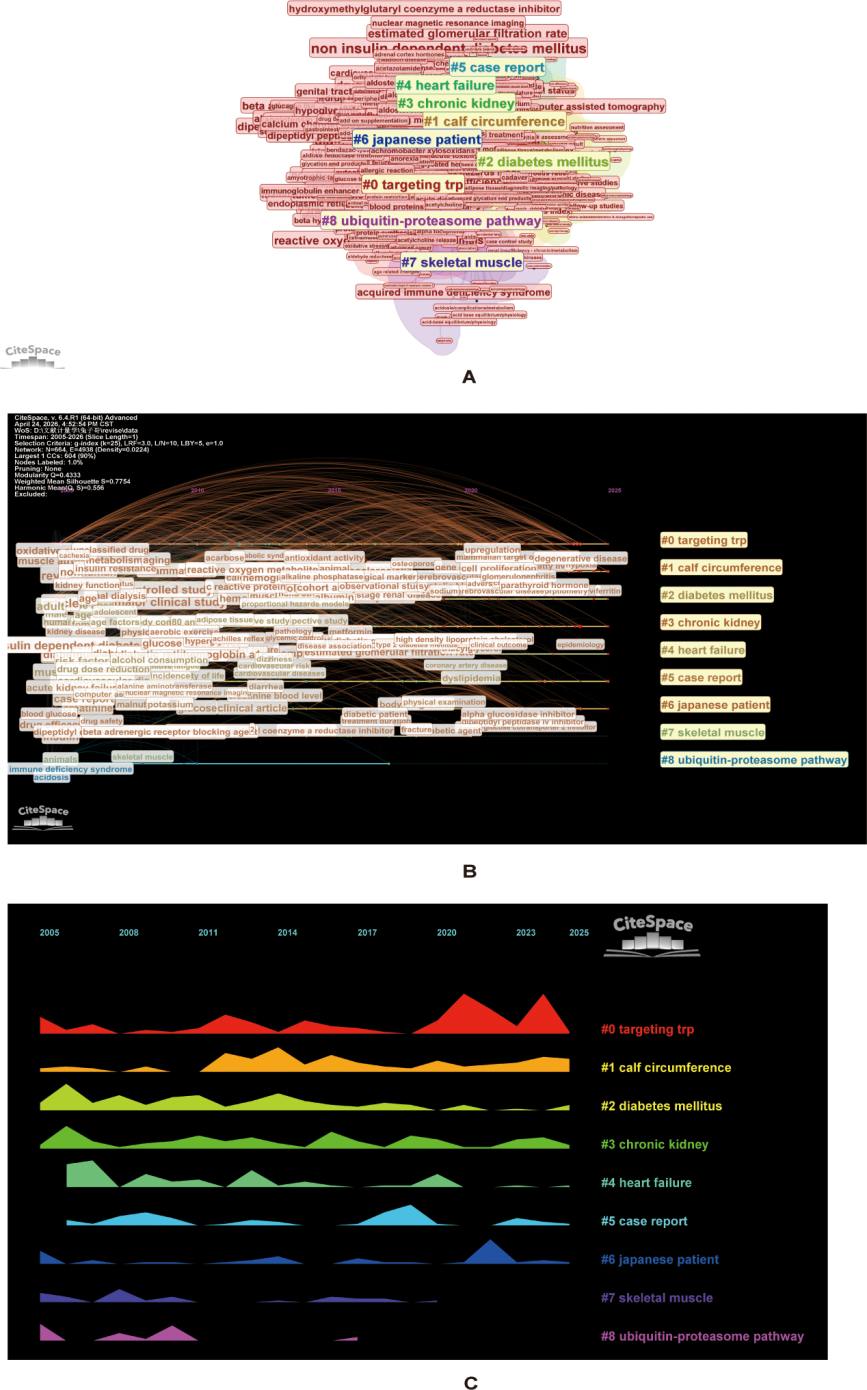
CiteSpace-based keyword clustering and temporal evolution analysis. **(A)** Keyword clustering map generated by CiteSpace. **(B)** Timeline view of keyword clusters, showing the temporal distribution and evolution of different research topics from 2005 to 2025. **(C)** Thematic evolution view of keyword clusters, illustrating changes in research attention over time and highlighting the transition from early clinical descriptors and renal dysfunction-related topics toward muscle function, metabolic pathways, and disease comorbidity.

**Figure 6:**
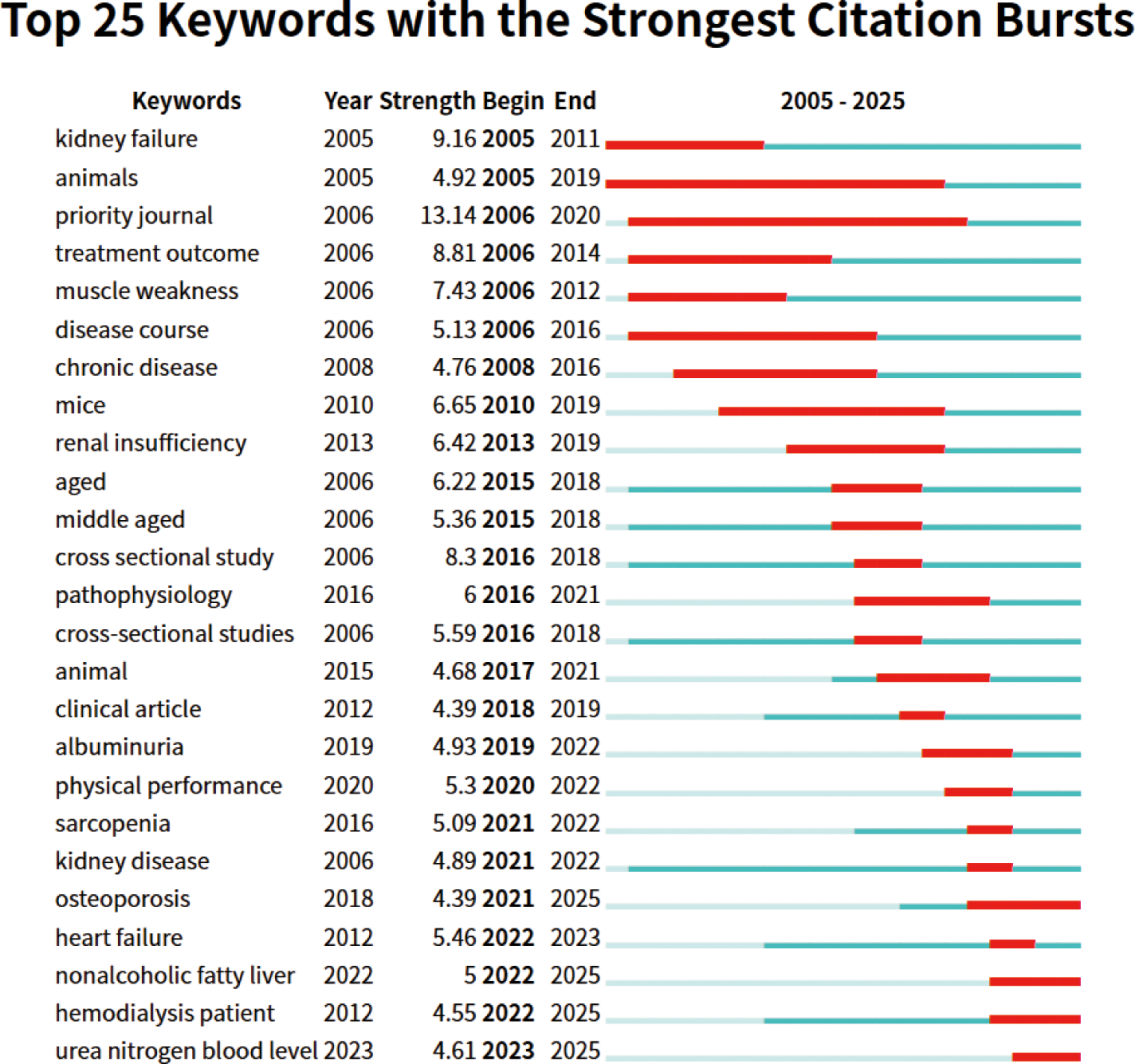
Top 25 keywords with the strongest burst intensity from 2005 to2025.

CiteSpace was further used for keyword clustering, timeline visualization, and burst term analysis to identify thematic evolution and research frontiers in this field (**Figures 5**, **6**). Keyword clustering identified the major clusters as #0 targeting trp, #1 calf circumference, #2 diabetes mellitus, #3 chronic kidney, #4 heart failure, #5 case report, #6 Japanese patient, #7 skeletal muscle, and #8 ubiquitin-proteasome pathway (**Figure 5A**). These clusters suggest that research themes in this field mainly focus on disease phenotypes, muscle function assessment, the relationship between diabetes and chronic kidney disease, cardiorenal-metabolic comorbidities, and mechanisms related to muscle protein degradation and signaling pathways.

The timeline visualization showed that early studies focused more on clinical outcomes and disease progression, including kidney failure, treatment outcome, muscle weakness, and disease course. Research then gradually expanded to disease phenotypes and muscle assessment indicators, such as diabetes mellitus, chronic kidney disease, calf circumference, and skeletal muscle. In recent years, mechanistic keywords related to targeting trp, ubiquitin-proteasome pathway, oxidative stress, and reactive oxygen species have become increasingly active. This suggests that the field is shifting from clinical observation and epidemiological association studies toward investigations of muscle protein metabolism, oxidative stress, and potential intervention targets (**Figures 5B, C**).

Burst keyword analysis showed that early burst terms mainly included kidney failure, treatment outcome, muscle weakness, and disease course, indicating that early research focused on renal failure status, treatment outcomes, and impaired muscle function. Mid-stage burst terms included renal insufficiency, cross sectional study, pathophysiology, and animal, suggesting a gradual expansion toward cross-sectional studies, pathophysiological mechanisms, and animal models. Recently persistent burst terms included osteoporosis, nonalcoholic fatty liver, hemodialysis patient, and urea nitrogen blood level, indicating that recent research hotspots have further extended to bone metabolism abnormalities, metabolic comorbidities, hemodialysis populations, and renal function-related indicators. These findings reflect a gradual shift in research on DKD and sarcopenia toward multisystem comorbidity, mechanistic exploration, and clinical risk stratification (**Figure 6**). It should be noted that CiteSpace cluster labels are automatically generated by the software based on keywords or textual terms. Some labels may mainly reflect combinations of high-frequency terms and should not be directly interpreted as independent, mature research directions or causal mechanisms.

### 3.6 Citation performance of the top 10 countries and journals

**Figure 7:**
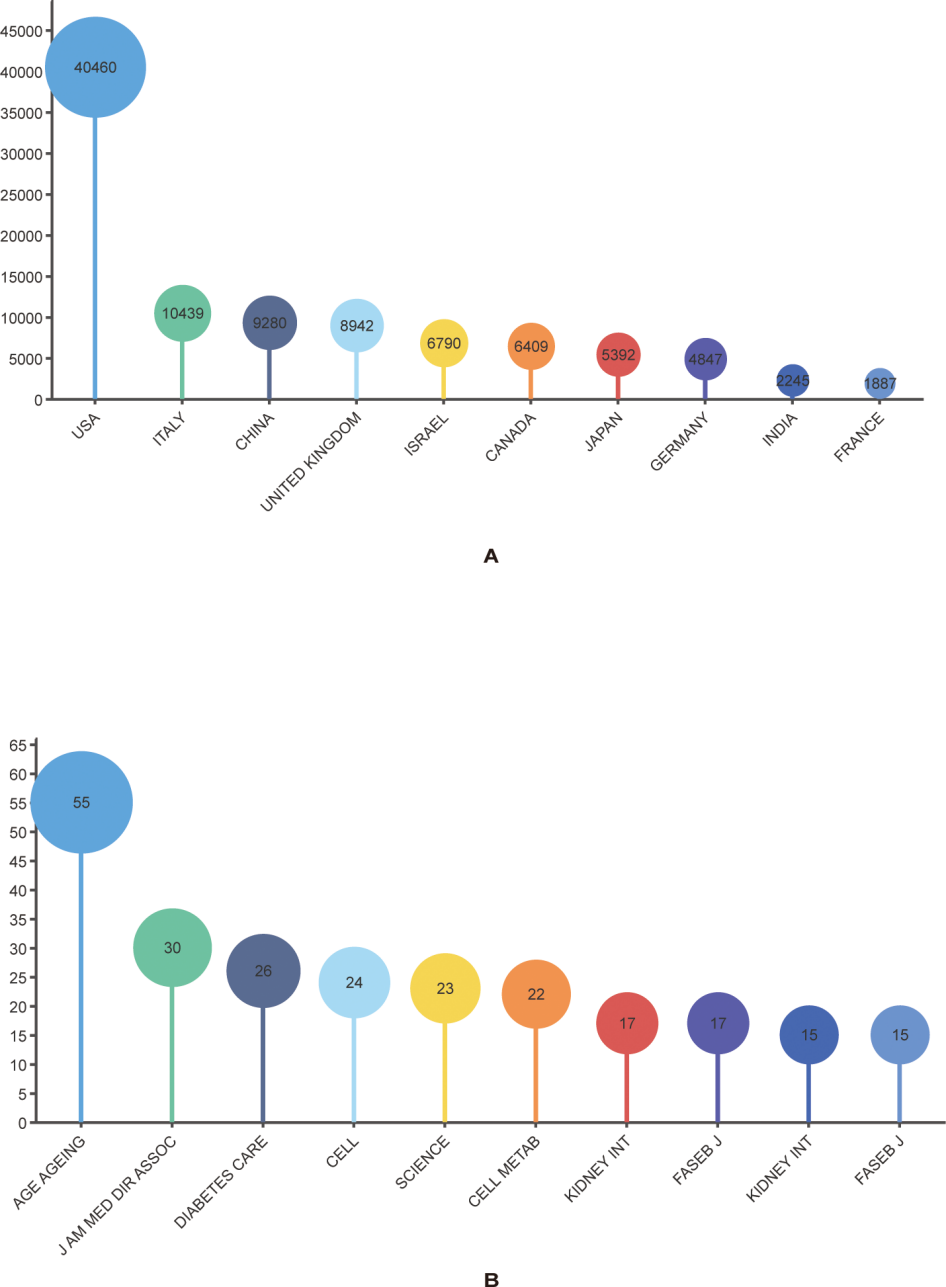
Citation performance of major countries/regions and journals. **(A)** Top 10 countries/regions ranked by total citation frequency. **(B)** Top 10 journals ranked by local citation frequency.

**Figure 7** presents the citation performance in this field, with **Figure 7A** showing total global citations at the country/region level and **Figure 7B** showing local citations of source journals within the present dataset. Overall, citation impact showed a clear pattern of concentration, with a small number of countries and journals occupying central positions in knowledge dissemination in this field.

At the country level, the United States had the highest total number of citations, reaching 40,460, far exceeding other countries and demonstrating its dominant academic influence in this research field. Italy ranked second with 10,439 total citations, followed by China with 9,280 citations. However, China had a relatively lower average citation count per publication, suggesting that although China has a high publication output, the international citation impact of its individual publications still has room for improvement. The United Kingdom, Israel, and Canada had total citation counts of 8,942, 6,790, and 6,409, respectively. Although Israel had a relatively small number of publications, its average citation count was high, suggesting strong impact per publication. Japan, Germany, India, and France also ranked among the top 10, indicating that research influence in this field is mainly concentrated in North America, Europe, and East Asia.

At the source journal level, Age and Ageing had the highest local citation count, with 55 citations, suggesting that research in geriatrics plays an important knowledge-base role in the intersecting topic of DKD and sarcopenia. Journal of the American Medical Directors Association and Diabetes Care ranked second and third, with 30 and 26 local citations, respectively, reflecting the central roles of geriatrics, diabetology, and chronic disease management research in this field. In addition, high-impact journals in basic life sciences and metabolic research, such as Cell, Science, and Cell Metabolism, also ranked among the leading journals, indicating that this topic is not only related to clinical disease management but is also closely associated with metabolic regulation, cellular signaling, and basic mechanistic research. The presence of Kidney International and FASEB Journal further indicates that nephrology, basic medicine, and translational medicine jointly constitute important knowledge sources in this field. Overall, **Figure 7** suggests that research on DKD and sarcopenia has strong interdisciplinary characteristics, with high-impact findings mainly distributed across geriatrics, diabetes and metabolism, nephrology, and basic life sciences.

### 3.7 Top 20 globally cited and locally cited publications

Citation analysis showed that globally highly cited publications were mainly concentrated in foundational and interdisciplinary areas, including inflammageing, diabetic complications, molecular mechanisms of skeletal muscle atrophy, mitochondrial dysfunction, and regulation of muscle mass (**Table 2**). The study by Ferrucci et al. on “inflammageing” ranked first with 2,706 global citations, suggesting that the associations among chronic inflammation, aging, cardiovascular disease, and frailty constitute an important theoretical background for this field. The study by Boulton et al. on the global burden of diabetic foot disease received 1,677 citations, reflecting the foundational role of research on diabetes-related complications in this field.

**Table 2:** Top 10 globally cited publications.

Number	First author	Article name	Source	Year	Global citations
1	Ferrucci L	Inflammageing: chronic inflammation in ageing, cardiovascular disease, and frailty	Nat Rev Cardiol	2018	2706
2	Boulton AJM	The global burden of diabetic foot disease	Diabetes Care	2005	1677
3	Gomes MD	Atrogin-1, a muscle-specific F-box protein highly expressed during muscle atrophy	Proc Natl Acad Sci U S A	2001	1433
4	Lecker SH	Multiple types of skeletal muscle atrophy involve a common program of changes in gene expression	FASEB J	2004	1255
5	Cohen S	Muscle wasting in disease: molecular mechanisms and promising therapies	Nat Rev Drug Discov	2015	896
6	Sandri M	PGC-1α protects skeletal muscle from atrophy by suppressing FoxO3 action and atrophy-specific gene transcription	Proc Natl Acad Sci U S A	2006	841
7	Wiley CD	The metabolic roots of senescence: mechanisms and opportunities for intervention	Nat Metab	2021	528
8	Sacheck JM	Rapid disuse and denervation atrophy involve transcriptional changes similar to those of muscle wasting during systemic diseases	FASEB J	2007	468
9	Smith RAJ	Mitochondria-targeted antioxidants in the treatment of disease	Trends Pharmacol Sci	2012	461
10	Sartori R	Smad2 and 3 transcription factors control muscle mass in adulthood	Am J Physiol Cell Physiol	2009	402
11	Romanello V	Mitochondrial quality control and muscle mass maintenance	Front Physiol	2016	372
12	Vinik AI	Diabetic neuropathies	Endocrinol Metab Clin North Am	2013	317
13	Horton WB	Microvascular dysfunction in diabetes mellitus and cardiometabolic disease	Endocr Rev	2021	285
14	Mammucari C	FoxO3 controls autophagy in skeletal muscle *in vivo*	Autophagy	2008	241
15	Goldberg EL	Drivers of age-related inflammation and strategies for healthspan extension	Immunol Rev	2015	223
16	Rom O	The role of E3 ubiquitin-ligases MuRF-1 and MAFbx in loss of skeletal muscle mass	Free Radic Biol Med	2016	218
17	Buford TW	Models of accelerated sarcopenia: critical pieces for solving the puzzle of age-related muscle atrophy	Ageing Res Rev	2010	217
18	Engelke K	Quantitative analysis of skeletal muscle by computed tomography imaging: state of the art	J Orthop Transl	2018	216
19	Dolly JO	The structure and mode of action of different botulinum toxins	Eur J Neurol	2006	204
20	Zhang H	Oxidative stress: roles in skeletal muscle atrophy	Biochem Pharmacol	2023	203

At the same time, classic studies by Gomes, Lecker, Cohen, Sandri, Sacheck, and others on muscle atrophy, atrogin-1, FoxO3, PGC-1α, and mechanisms of disease-related muscle wasting also showed high global influence. This indicates that research on chronic kidney disease/DKD and sarcopenia relies heavily on mechanistic literature related to skeletal muscle metabolism, protein degradation, and maintenance of muscle function.

In comparison, locally cited publications more directly reflect knowledge connections within the included dataset. Studies by Lecker, Gomes, Sacheck, Cohen, and Sandri ranked among the top locally cited publications, with 15, 14, 10, 9, and 7 local citations, respectively (**Table 3**). This further indicates that common transcriptional programs of muscle atrophy, the ubiquitin-proteasome system, FoxO-related pathways, and disease-related muscle wasting represent repeatedly cited core theoretical foundations in this research direction.

**Table 3:** Top locally cited publications.

Number	First author	Article Name	Source	Year	Local citations	Global citations	LC/GC ratio (%)
1	Lecker SH	Multiple types of skeletal muscle atrophy involve a common program of changes in gene expression	FASEB Journal	2004	15	1255	1.20
2	Gomes MD	Atrogin-1, a muscle-specific F-box protein highly expressed during muscle atrophy	Proceedings of the National Academy of Sciences of the United States of America	2001	14	1433	0.98
3	Sacheck JM	Rapid disuse and denervation atrophy involve transcriptional changes similar to those of muscle wasting during systemic diseases	FASEB Journal	2007	10	468	2.14
4	Cohen S	Muscle wasting in disease: molecular mechanisms and promising therapies	Nature Reviews Drug Discovery	2015	9	896	1.00
5	Sandri M	PGC-1α protects skeletal muscle from atrophy by suppressing FoxO3 action and atrophy-specific gene transcription	Proceedings of the National Academy of Sciences of the United States of America	2006	7	841	0.83
6	Fung FY	Prevalence of and factors associated with sarcopenia among multi-ethnic ambulatory older Asians with type 2 diabetes mellitus in a primary care setting	BMC Geriatrics	2019	5	70	7.14
7	Ida S	Association between sarcopenia and renal function in patients with diabetes: a systematic review and meta-analysis	Journal of Diabetes Research	2019	5	40	12.50
8	Çeliker M	The relationship between sarcopenia and DKD in patients with type 2 diabetes mellitus	Romanian Journal of Internal Medicine	2018	5	26	19.23
9	Feng LY	Prevalence and risk factors of sarcopenia in patients with diabetes: a meta-analysis	Journal of Clinical Endocrinology & Metabolism	2022	4	174	2.30
10	Sartori R	Smad2 and 3 transcription factors control muscle mass in adulthood	American Journal of Physiology-Cell Physiology	2009	3	402	0.75
11	Zhang H	Skeletal muscle atrophy in diabetic kidney disease: mechanisms and potential therapeutic strategies	Biochemical Pharmacology	2023	3	203	1.48
12	Tang G	Butyrate ameliorates skeletal muscle atrophy in DKD by enhancing gut barrier function and FFA2-mediated PI3K/Akt/mTOR signals	British Journal of Pharmacology	2022	3	182	1.65
13	Huang YM	Association between sarcopenia and DKD in patients with type 2 diabetes mellitus	Journal of Clinical Medicine	2022	3	14	21.43
14	Wang M	Mechanisms of skeletal muscle atrophy in patients with diabetes mellitus	Frontiers in Endocrinology	2020	2	197	1.02
15	Purnamasari D	The emergence of sarcopenia in patients with diabetes mellitus: current evidence and future direction	Review of Diabetic Studies	2022	2	93	2.15
16	Ogama N	Association between sarcopenia and DKD in patients with type 2 diabetes mellitus	Journal of Clinical Medicine	2019	2	60	3.33
17	Karakousis ND	The relationship between sarcopenia and proteinuria in patients with chronic kidney disease	International Urology and Nephrology	2023	2	15	13.33
18	Romanello V	Mitochondrial quality control and muscle mass maintenance	Frontiers in Physiology	2016	1	372	0.27
19	Mammucari C	FoxO3 controls autophagy in skeletal muscle *in vivo*	Autophagy	2008	1	241	0.41
20	Goldberg EL	Drivers of age-related inflammation and strategies for healthspan extension	Immunological Reviews	2015	1	223	0.45

LC/GC ratio (%), local citations/global citations × 100.

It is noteworthy that some studies with relatively modest global citation counts but high local citation proportions, such as those by Çeliker, Ida, Huang, and Karakousis, mainly focused on the clinical associations of sarcopenia with DKD renal function decline, proteinuria, or chronic kidney disease. This suggests that although these publications may have limited overall academic influence, they have strong specificity and field relevance within the present research topic. Overall, globally highly cited publications mainly reflect the broad theoretical background and mechanistic basis on which this field relies, whereas locally highly cited publications more specifically reflect the core knowledge sources and thematic evolution of the intersection between chronic kidney disease/DKD and sarcopenia.

### 3.8 Disease-related gene intersection analysis between DKD and sarcopenia

**Figure 8:**
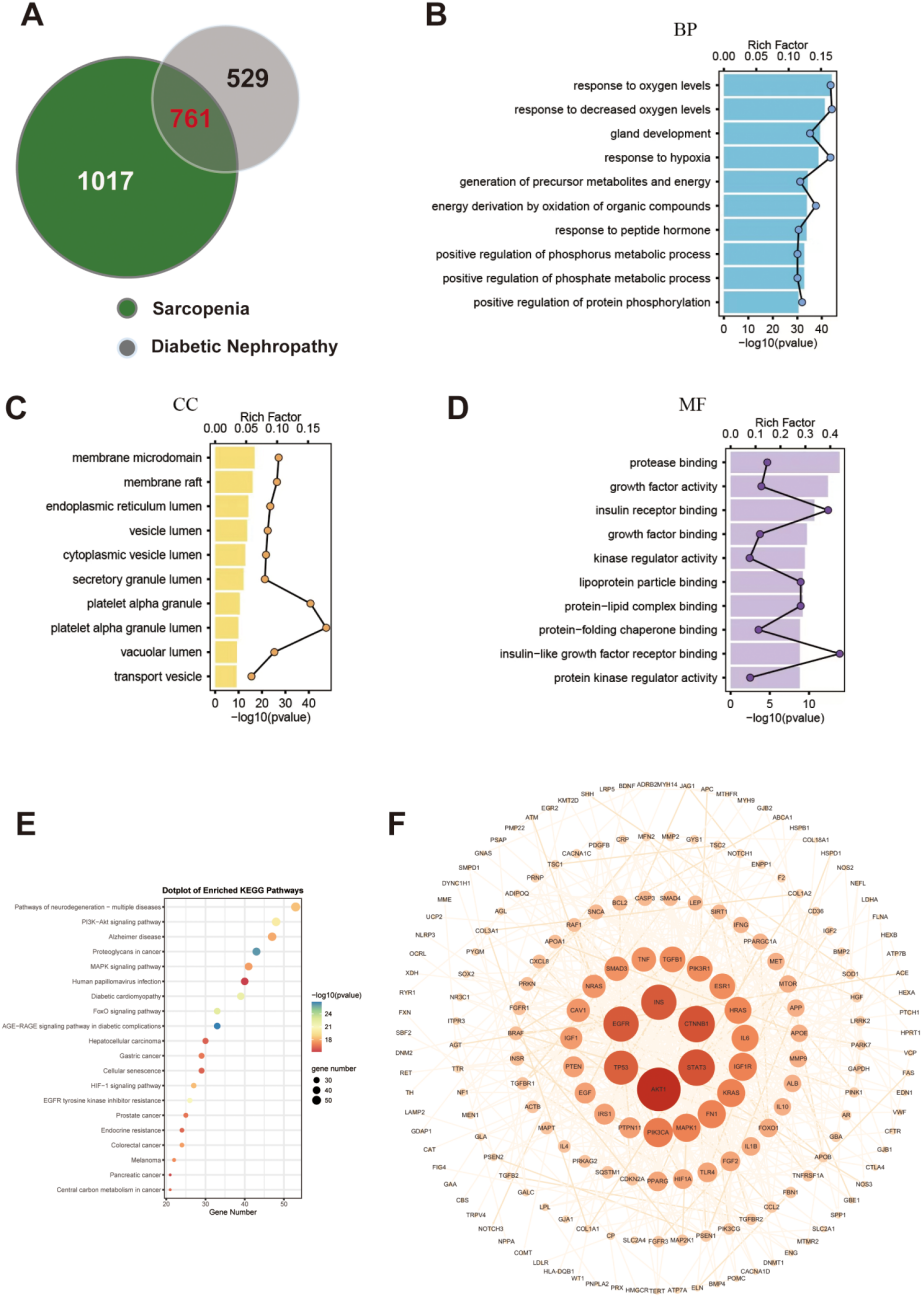
Genetic overlap and functional enrichment analysis of diabetic kidney disease and sarcopenia. **(A)** Venn diagram showing the overlap between sarcopenia-related genes and diabetic kidney disease-related genes. **(B–D)** Gene Ontology enrichment analysis of the shared genes, including biological process **(B)**, cellular component **(C)**, and molecular function **(D)**. **(E)** KEGG pathway enrichment analysis of shared genes. The enriched pathways included PI3K-Akt signaling pathway. **(F)** Gene-pathway interaction network.

To conduct an exploratory analysis of potential molecular intersections between sarcopenia and DKD, we performed an hypothesis-generating cross-disease gene overlap analysis. As shown in **Figure 8A**, 1,017 genes were specific to the sarcopenia-related comparison, 529 genes were specific to the DKD-related comparison, and 761 genes overlapped between the two gene lists. These overlapping genes were considered cross-disease candidate genes for subsequent enrichment and network analyses. Importantly, this overlap analysis only indicates that these genes appeared in both disease-related gene sets at the database level; it does not provide information regarding the direction, magnitude, tissue specificity, or causal role of gene expression changes, nor does it imply that these genes are differentially expressed or mechanistically involved in either disease.

GO enrichment analysis was then performed to characterize the biological functions potentially associated with the overlapping candidate genes. In the biological process category, the enriched terms were mainly related to response to oxygen levels, response to decreased oxygen levels, response to hypoxia, generation of precursor metabolites and energy, energy derivation by oxidation of organic compounds, response to peptide hormone, and positive regulation of protein phosphorylation (**Figure 8B**). These results suggest that hypoxia response, energy metabolism, peptide hormone signaling, and phosphorylation-related regulation may represent common biological themes within the overlapping gene set at the database annotation level.

For the cellular component category, the enriched terms included membrane microdomain, membrane raft, endoplasmic reticulum lumen, vesicle lumen, cytoplasmic vesicle lumen, secretory granule lumen, platelet alpha granule, and transport vesicle (**Figure 8C**). These findings show that the overlapping genes were enriched in membrane-associated signaling structures, vesicle-related compartments, and secretory or transport-related cellular components. For the molecular function category, the major enriched terms included protease binding, growth factor activity, insulin receptor binding, growth factor binding, kinase regulator activity, lipoprotein particle binding, protein–lipid complex binding, and insulin-like growth factor receptor binding (**Figure 8D**). These molecular functions are consistent with biological processes involving metabolic regulation, growth factor signaling, proteolytic regulation, and kinase-mediated signal transduction.

KEGG pathway enrichment analysis further showed that the overlapping candidate genes were enriched in several signaling and disease-related pathways, including the PI3K-Akt signaling pathway, MAPK signaling pathway, AGE-RAGE signaling pathway in diabetic complications, FoxO signaling pathway, HIF-1 signaling pathway, diabetic cardiomyopathy, and cellular senescence (**Figure 8E**). These pathways may be implicated in metabolic stress, inflammation, oxidative stress, hypoxia adaptation, insulin signaling, and cell survival. However, several enriched KEGG terms, such as cancer- and neurodegeneration-related pathways, should be interpreted cautiously because they may reflect shared signaling architecture and database annotation density rather than disease-specific mechanisms of DKD–sarcopenia comorbidity. Furthermore, pathway enrichment results should be regarded as database-level associations rather than evidence of active pathway involvement in disease pathogenesis.

A protein–protein interaction network was constructed to examine the interaction structure among the overlapping candidate genes (**Figure 8F**). Several highly connected nodes, including AKT1, STAT3, TP53, CTNNB1, EGFR, INS, IL6, TNF, VEGFA, MAPK1, FN1, PPARG, and CASP3, appeared near the center of the network. These genes are widely associated with inflammatory signaling, insulin and growth-factor signaling, cellular stress responses, apoptosis, extracellular matrix remodeling, and metabolic regulation. Nevertheless, because many of these genes are broadly connected signaling hubs implicated in multiple chronic diseases, their network centrality should not be interpreted as evidence that they are specific drivers of the DKD–sarcopenia axis. Instead, these hub genes should be regarded as network-level candidate molecules that may help generate hypotheses for future validation.

Overall, this exploratory analysis suggests that the molecular overlap between sarcopenia and DKD may be related to hypoxia response, energy metabolism, insulin/growth-factor signaling, inflammatory regulation, cellular stress, and extracellular matrix-associated pathways. These findings provide candidate molecular themes that are broadly consistent with the clinical features of renal dysfunction, metabolic disturbance, muscle wasting, and functional decline. However, the present analysis remains strictly hypothesis-generating and is based solely on database mining without experimental validation or directional expression information. Further transcriptomic validation, direction-specific differential expression analysis, tissue- or cell-type-specific omics studies, and functional experiments are required to determine whether these candidate genes and pathways are mechanistically involved in DKD–sarcopenia comorbidity.

## 4 Discussion

### 4.1 Epidemiological burden: bibliometric trends and the paradigm shift toward functional outcomes

Based on three databases, including WoSCC, Scopus, and PubMed, this study systematically mapped the research landscape, knowledge structure, and thematic evolution of the intersection between DKD and sarcopenia. Overall, the number of publications in this field showed a gradual upward trend, indicating that the relationship between diabetes-related kidney injury and reduced skeletal muscle mass, decreased muscle strength, and impaired physical function has received increasing attention. DKD is not only an important cause of progression from diabetes to chronic kidney disease and end-stage kidney disease, but also an important clinical condition that accelerates systemic metabolic disorders, malnutrition, inflammatory activation, and functional decline. Meanwhile, sarcopenia is common among patients with chronic kidney disease and is associated with falls, frailty, hospitalization, reduced quality of life, and increased mortality risk. Recent systematic reviews and meta-analyses on the prevalence of sarcopenia in patients with chronic kidney disease also suggest that this problem represents a substantial global disease burden (5, 18).

From the bibliometric results, the growth of research on the intersection between DKD and sarcopenia is not accidental, but is closely related to global population aging, the increasing burden of diabetes and chronic kidney disease, and the shift in research focus from “renal outcomes” to “functional outcomes” and “patient-centered outcomes.” Previous studies on DKD have mainly focused on hard endpoints such as proteinuria, decline in estimated glomerular filtration rate, kidney failure, cardiovascular events, and mortality. In recent years, however, indicators such as muscle mass, muscle strength, gait speed, physical activity capacity, nutritional status, and frailty have gradually entered the research field, reflecting a transition from single-organ injury toward multisystem functional decline (19). This change is reflected in international guidance. ADA 2026 highlights both cardiorenal protection and functional status, whereas EWGSOP2 adopts physical performance measures like gait speed as central to sarcopenia diagnosis.

### 4.2 Clinical phenotype overlap: diagnostic pitfalls and implications for clinical management

DKD and sarcopenia show substantial overlap in clinical phenotypes. DKD diagnosis follows KDIGO 2024 CKD and ADA 2026 guidelines, using key indicators including eGFR (non-race-adjusted CKD-EPI) and UACR (20, 21). Sarcopenia diagnosis adheres to the EWGSOP2 consensus definition, starting with reduced muscle strength (men <27 kg, women <16 kg) and confirmed by impaired physical performance (4-meter walking speed ≤0.8 m/s) or low muscle mass (ASMI by DXA: men <7.0 kg/m², women <5.5 kg/m²) (22). These conditions likely interact through common mechanisms, including chronic inflammation, insulin resistance, nutritional deficiencies, metabolic disturbances, and reduced physical activity, which increase susceptibility to muscle loss and functional decline (23, 24).

Among DKD patients, sarcopenia-related phenotypes often overlap with protein-energy wasting, obesity, including sarcopenic obesity, frailty, and persistent inflammation (25). Traditional BMI assessments may be skewed by edema and fat deposits, implying that “stable weight” may not accurately reflect “stable muscle condition.” KDIGO 2024 guidelines emphasize lifestyle management, including dietary interventions, within their comprehensive CKD care model and recommend validated malnutrition assessment tools (e.g., SGA) (20). Protein-Energy Wasting (PEW), as defined by the International Society of Renal Nutrition and Metabolism, is depletion of protein and energy stores. It remains primarily a hypothesis-driven concept in routine practice and has yet to be incorporated into official KDIGO or ADA diagnostic frameworks. Diagnosis typically requires evaluation of biochemical indicators, weight or fat loss, insufficient protein/energy intake, and reduced muscle mass. Inflammatory markers may indicate risk but are not diagnostic alone (26).

This clinical phenotype overlap has important implications. First, it suggests that the prognostic risk of patients with DKD may be underestimated, because sarcopenia, frailty, and protein-energy wasting reflect systemic functional reserve beyond traditional renal indicators (27). Second, it indicates that clinical management should extend from simple “disease control” to “functional preservation,” meaning that muscle mass, muscle strength, nutritional status, body composition, and physical activity level should be systematically assessed while controlling blood glucose, blood pressure, proteinuria, and cardiorenal risk (28). Finally, it also suggests that future studies should include sarcopenia-related outcomes such as handgrip strength, gait speed, body composition, nutritional indicators, and physical performance in DKD cohorts and interventional studies, so as to more comprehensively evaluate disease burden and treatment benefit (29). It is noteworthy that, while current KDIGO and ADA guidelines do not yet recommend mandatory sarcopenia screening for DKD patients, an increasing body of evidence supports early detection in high-risk groups (30, 31).

### 4.3 Shared pathophysiology: common mechanisms and therapeutic implications

DKD and sarcopenia may share common pathophysiological foundations. However, it should be explicitly noted that current investigations into these mechanisms are mainly based on experimental research and observational data, aligning with hypothesis-driven studies, and concrete guidelines for pharmacological interventions have not yet been developed.

Long-term hyperglycemia and insulin resistance can not only promote glomerular hyperfiltration, oxidative stress, chronic inflammatory responses, and renal fibrosis, but also impair skeletal muscle responsiveness to insulin, affecting glucose uptake, protein synthesis, and mitochondrial function (32). In patients with type 2 diabetes, insulin resistance, chronic low-grade inflammation, accumulation of advanced glycation end products, and increased oxidative stress may adversely affect skeletal muscle mass, muscle strength, and physical function, thereby increasing the risk of sarcopenia (29). Meanwhile, decreased muscle mass and muscle function may further reduce glucose utilization, decrease physical activity, and aggravate metabolic disturbance, suggesting a potentially mutually reinforcing pathological cycle between diabetes and sarcopenia (33). During the progression of DKD, chronic decline in renal function may further amplify the above muscle injury processes. Uremic toxin accumulation, metabolic acidosis, chronic inflammation, anemia, abnormal vitamin D metabolism, insufficient nutritional intake, and reduced physical activity may all promote skeletal muscle protein degradation, inhibit muscle synthesis, and ultimately lead to reduced muscle mass and physical function decline (34). Therefore, DKD and sarcopenia may not simply coexist, but may jointly constitute a continuous pathological process along a “metabolic–inflammatory–kidney–muscle” axis.

This shared pathophysiological basis also helps explain the keyword and thematic clustering results of the present study. Recurrent themes such as inflammation, oxidative stress, insulin resistance, malnutrition, physical function, and chronic kidney disease suggest that the research focus in this field has gradually shifted from merely describing prevalence toward explaining why patients with DKD are more susceptible to muscle loss and functional decline (32, 35). However, it should be emphasized that bibliometric analysis reveals research hotspots, knowledge structures, and thematic evolution trends, rather than directly inferring causal chains.

From a clinical perspective, shared pathophysiological mechanisms suggest that DKD-associated sarcopenia may require multi-target intervention. Simply improving blood glucose control or providing nutritional supplementation alone may be insufficient to reverse muscle deterioration. A more reasonable strategy may need to simultaneously focus on metabolic control, inflammation modulation, renal protection, nutritional support, and exercise intervention. Previous reviews on CKD-related sarcopenia have also emphasized that combined interventions such as nutritional support and resistance exercise may help improve muscle mass, muscle strength, and physical function (36).

### 4.4 Molecular mechanisms: convergent pathways and pathogenic complexity

Hypergeometric analysis (p < 0.001) showed that gene overlap was greater than expected by chance, suggesting a potential shared molecular basis for DKD and sarcopenia. Functional enrichment analysis suggested that these overlapping genes may be associated with various biological processes, including inflammation, oxidative stress, insulin/IGF-1 signaling, hypoxia response, and energy metabolism. These results are exploratory and hypothesis-generating, providing potential molecular links into this comorbidity.From a systems biology perspective, key hub genes such as TP53 and STAT3 exhibit high network centrality, potentially serving as pleiotropic regulatory nodes influencing both renal and muscle pathologies. For instance, TP53 contributes to renal tubular epithelial cell apoptosis and fibrosis, and its elevated activity may relate to age-associated muscle loss (37). STAT3 is upregulated under hyperglycemic conditions and is also implicated in muscle atrophy. Through the JAK-STAT pathway, STAT3 may act as a molecular bridge between these conditions, partly supporting the hypothesis of shared regulatory mechanisms (38, 39).

DKD-associated sarcopenia may not be caused by nutritional deficiency alone, but may be related to a persistent imbalance between reduced muscle protein synthesis and enhanced protein degradation under CKD conditions. Metabolic acidosis, insulin resistance, chronic inflammation, uremic toxins, and hormonal imbalances jointly activate multiple catabolic pathways.Among them, impaired insulin/IGF-1–PI3K–Akt signaling can lead to activation of FoxO transcription factors, thereby promoting muscle protein degradation mediated by the ubiquitin–proteasome system and the autophagy–lysosome pathway. Inflammatory factors such as interleukin-6 and tumor necrosis factor-α can further amplify muscle catabolic responses and accelerate protein degradation by activating pathways such as myostatin signaling. Potential biomarkers such as Klotho, myostatin, and inflammatory factors also provide new research directions for the identification and monitoring of CKD-related sarcopenia (40, 41). Because systemic protein turnover is high, even a slight but persistent imbalance between protein synthesis and degradation may gradually lead to substantial muscle protein loss during long-term CKD and increase the risk of adverse outcomes. Therefore, DKD-associated sarcopenia should be understood as a complex syndrome jointly driven by metabolic abnormalities, inflammatory responses, uremic milieu, hormonal signaling disturbances, and activation of protein degradation pathways, rather than being explained solely by reduced muscle mass or insufficient nutritional intake (40, 42, 43).

In addition, diabetes itself may affect skeletal muscle structure and function through mechanisms such as glucotoxicity, lipotoxicity, accumulation of advanced glycation end products, microvascular injury, and neuropathy (23). For patients with DKD, these diabetes-related factors may overlap with chronic kidney disease-related factors, forming a more complex muscle injury environment (44). For example, insulin resistance is not only an important metabolic background for the progression of DKD, but also an important driver of reduced muscle protein synthesis and decreased muscle strength. Chronic inflammation can promote renal fibrosis and also enhance muscle catabolism through pathways such as NF-κB, TNF-α, and IL-6 (45–47).

It is worth noting that current evidence regarding the intersecting mechanisms between DKD and sarcopenia remains limited. Many molecular pathways are mainly derived from studies of chronic kidney disease, diabetes, or sarcopenia as separate fields, and there is still a lack of mechanistic validation specifically targeting patients with both DKD and sarcopenia (24, 48). Therefore, in the discussion of the present study, these molecular mechanisms are more appropriately interpreted as biological background for explaining the literature clustering results, rather than as mechanisms directly demonstrated by this study. Currently, neither the KDIGO, ADA, nor the EWGSOP2 guidelines have recommended any molecular biomarkers into routine clinical practice. This underscores the lengthy translational process from mechanistic discoveries to clinical application, highlighting the need for further clinical validation studies to substantiate the practical utility of these targets.

### 4.5 Multi-omics insights: molecular crosstalk and translational implications in DKD-sarcopenia

Omics research provides a new mechanistic perspective for understanding the potential links between DKD and sarcopenia. Traditional studies have mostly relied on clinical indicators, inflammatory factors, or single biomarkers, whereas transcriptomics, proteomics, metabolomics, and multi-omics integration can identify potential molecular interactions among the kidney, blood circulation, and skeletal muscle at the systems level. In recent years, some studies have attempted to explore a potential “kidney-muscle crosstalk” network for CKD-related sarcopenia using multi-omics strategies.

For example, For example, an adenine-induced CKD mouse model integrated multi-omics (kidney, serum, muscle) to identify key molecules that may mediate CKD-related muscle atrophy. Among these molecules, Spp1 was found to be significantly increased in both the kidneys and serum of CKD mice and may affect skeletal muscle through the circulation. Further *in vitro* experiments showed that recombinant Spp1 promoted C2C12 myotube atrophy and upregulated the muscle atrophy marker Murf-1. Animal experiments also showed that pharmacological inhibition of Spp1 increased the weights of the gastrocnemius and tibialis anterior muscles and improved the muscle atrophy phenotype. Transcriptomic analysis further suggested that differentially expressed genes in skeletal muscle after Spp1 inhibition were mainly enriched in protein digestion and absorption, the glucagon signaling pathway, the apelin signaling pathway, and extracellular matrix–receptor interaction pathways.

These findings suggest that CKD-related sarcopenia may not be caused simply by malnutrition, reduced physical activity, or uremic toxin accumulation, but may be subject to systemic regulation mediated by kidney-derived secretory factors. Since DKD is an important cause of CKD, similar kidney–muscle molecular crosstalk mechanisms may also participate in the development and progression of sarcopenia in patients with DKD, supporting the plausibility of the thematic evolution of the “metabolic–inflammatory–kidney–muscle axis” observed in the present study (49, 50).

For the intersection between DKD and sarcopenia, the value of omics research is mainly reflected in three aspects. It helps identify key pathways linking kidney injury and muscle degeneration, including inflammation, energy metabolism, mitochondrial function, protein degradation, and extracellular matrix remodeling, and facilitates discovery of potential biomarkers for early detection of high-risk patients, beyond late manifestations like muscle loss. It also provides molecular perspectives into why patients with similar renal function may exhibit varying degrees of muscle deterioration and functional impairment (35, 41, 51). However, the interpretation of omics results also requires caution. It should be emphasized that no omics-based biomarkers are currently recommended by the KDIGO, ADA, or EWGSOP2 guidelines for clinical diagnosis or therapeutic decision-making. Different databases, sample sources, tissue types, sequencing platforms, and statistical thresholds may significantly influence the selection of candidate genes or pathways. Without independent cohort validation, experimental verification, or clinical phenotype association, differentially expressed genes or enriched pathways alone cannot be directly translated into reliable diagnostic or therapeutic targets. Therefore, future studies should strengthen multicenter sampling, longitudinal data collection, external validation, and functional experimental verification to improve the reproducibility and clinical interpretability of omics discoveries (52).

### 4.6 Therapeutic targets: current interventions and future directions

Management of DKD concomitant with sarcopenia requires a clear distinction between evidence-based practices and emerging concepts. Current guidelines highlight holistic DKD management, including glycemic control and blood pressure regulation. Evidence-based pharmacological interventions recommended by ADA 2026 and KDIGO 2024 include SGLT2 inhibitors, non-steroidal MRAs, GLP-1 receptor agonists, and ACEIs/ARBs, which help delay renal function decline, mitigate cardiovascular risks, and improve metabolic health (20, 21). Nevertheless, no pharmacological treatments for sarcopenia have been approved, and evidence is insufficient to show direct improvement in muscle mass, strength, or physical performance. Thus, traditional renoprotective or glucose-lowering treatments alone may be inadequate to address muscle atrophy in DKD patients.

Non-pharmacological strategies remain core approaches for sarcopenia management (53). ADA 2026 guidelines advise daily protein intake of 0.8 g/kg (Grade A) for CKD G3+ non-dialysis patients and 1.0–1.2 g/kg (Grade B) for dialysis patients (21). The AWGS notes that targeted protein supplementation may be required, highlighting the clinical challenge of balancing restriction with supplementation, emphasizing the need to optimize muscle protein synthesis while ensuring renal protection. Resistance training enhances muscle strength and physical performance (53), addressing protein substrate deficits caused by dietary restrictions in DKD patients.

Emerging therapeutic targets, such as myostatin inhibitors and Klotho supplementation (54, 55), remain hypothesis-driven and far from guideline inclusion. Although promising in isolated diseases, they are not approved or recommended for DKD patients with sarcopenia. Current research hotspots focus on inflammatory pathways, oxidative stress, and mitochondrial dysfunction, offering directions for future exploration. Advances in omics and mechanistic studies suggest some molecular targets or biomarkers may support early detection, risk stratification, and individualized intervention in DKD-associated sarcopenia. Proposed targets require multi-level validation through mechanistic studies, animal models, clinical cohorts, and intervention trials. Bibliometric analysis can identify trends, gaps, and potential hotspots but cannot directly demonstrate therapeutic efficacy. Future studies should combine longitudinal cohorts, multi-omics, functional studies, and RCTs to clarify modifiable components and advance precision prevention and treatment.

### 4.7 Clinical trials: evidence gaps and future research priorities

Although both DKD and sarcopenia have clear clinical importance, high-quality clinical trials targeting the intersection of these two populations remain insufficient. Existing clinical trials on DKD mostly use renal outcomes, cardiovascular outcomes, glycemic control, or drug safety as primary endpoints, while relatively few systematically evaluate functional outcomes such as muscle mass, muscle strength, gait speed, physical performance, frailty status, and quality of life. Conversely, intervention studies related to sarcopenia have mainly focused on general older adults, patients with chronic kidney disease, or dialysis patients, and evidence specifically targeting patients with DKD complicated by sarcopenia remains limited.

Existing studies on CKD-related sarcopenia suggest that sarcopenia is relatively common among patients with CKD, and its occurrence may be associated with uremic toxin accumulation, chronic inflammation, malnutrition, sedentary behavior, reduced muscle protein synthesis, and enhanced catabolism. A scoping review showed that the prevalence of sarcopenia in patients with CKD varies widely depending on diagnostic criteria, study population, and disease stage, and is associated with adverse outcomes such as disability, hospitalization, and mortality (18). However, although some randomized controlled trials have evaluated the effects of exercise, nutrition, or combined interventions on muscle mass, muscle strength, and physical function in patients with CKD, studies that clearly target “CKD complicated by sarcopenia” as the specific population remain insufficient.

Existing intervention research primarily focuses on exercise training and nutritional management, yet both face significant challenges in evidence synthesis and clinical translation. Regarding exercise interventions, resistance training has been shown to improve muscle strength in CKD patients; however, heterogeneity in inclusion criteria, intervention methods, intensity, adherence, and diagnostic standards hampers evidence synthesis, and the lack of standardized protocols limits broader clinical adoption (56). Nutritional interventions require delicate balancing: overly strict protein restriction may accelerate muscle wasting, whereas excessive intake can increase renal metabolic burden. Current evidence is insufficient to define optimal protein intake for DKD patients, and personalized dietary recommendations require validation in robust randomized controlled trials (RCTs). Future studies should avoid directly applying nutritional strategies designed for general elderly or CKD populations; instead, individualized dietary regimens should be tailored according to the patient’s eGFR, proteinuria level, dialysis status, glycemic control, nutritional risk, and severity of sarcopenia.

At present, there is no established consensus for the design of clinical trials targeting DKD with concomitant sarcopenia. In light of this, the following exploratory directions are proposed. Future research should prioritize high-risk subgroups and incorporate EWGSOP2 core indicators (such as handgrip strength, gait speed, and SPPB scores) as secondary outcomes to evaluate the effects of standard cardio-renal protective therapies, including SGLT2 inhibitors and finerenone, on muscle health. Secondly, precise stratification based on eGFR, proteinuria, sarcopenia severity, and nutritional risk is essential to identify subgroups most likely to benefit. Third, multidimensional integrated approaches that combine cardio-renal protective drugs, personalized nutrition, and resistance training should be explored to provide robust evidence to inform guideline updates. Lastly, drug effect evaluations should extend to functional outcomes, emphasizing the potential impact of agents such as SGLT2 inhibitors on muscle function, thereby generating a more comprehensive evidence base for their clinical value.

### 4.8 Precision medicine perspective: a three-stage integrated management framework

**Figure 9:**
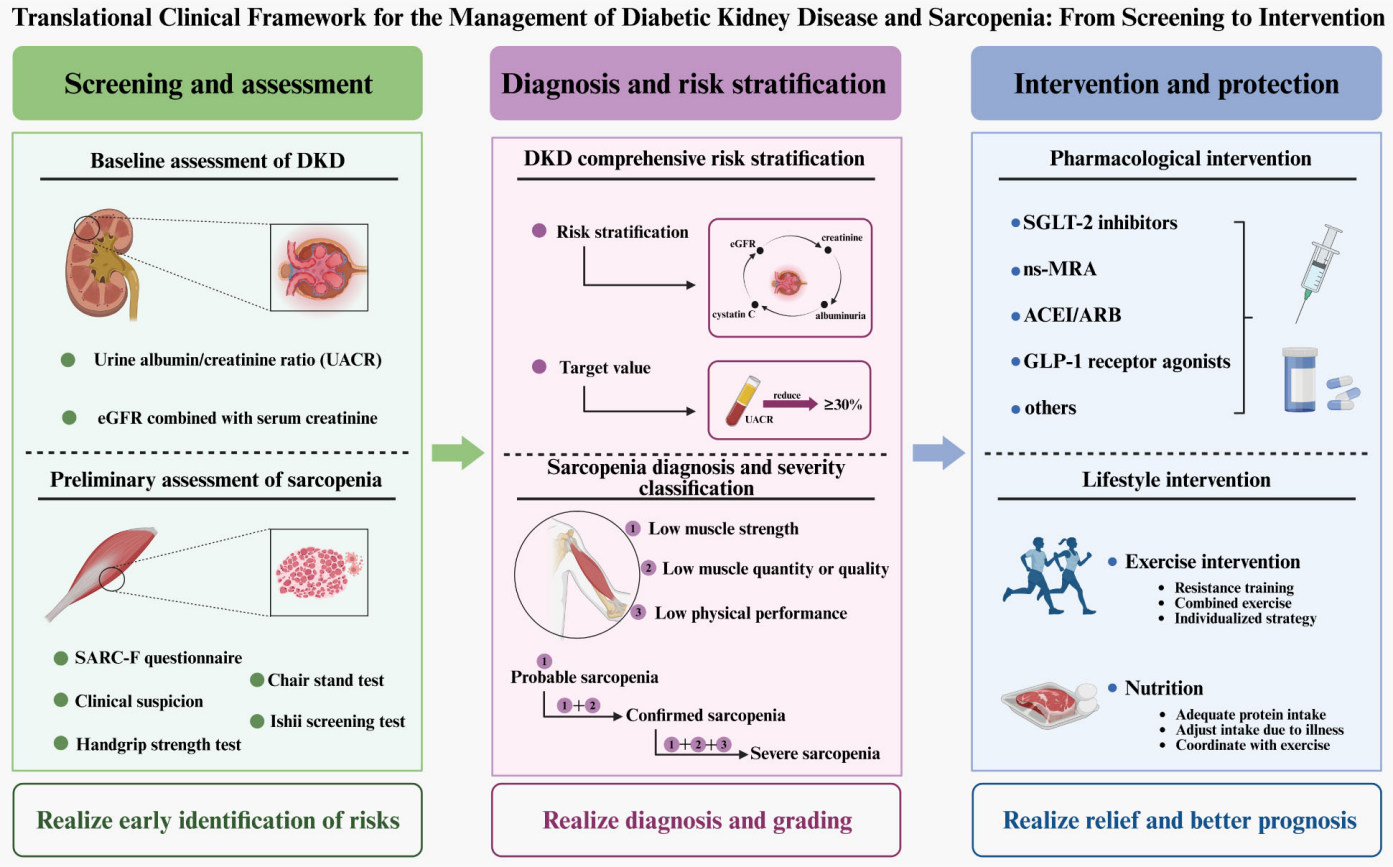
Translational clinical framework for the management of diabetic kidney disease and sarcopenia: from screening to intervention.

From a precision medicine perspective, DKD complicated by sarcopenia is not a homogeneous population. Patients differ substantially in age, sex, diabetes duration, renal function stage, albuminuria level, nutritional status, inflammatory burden, physical activity level, degree of obesity, and comorbidities. These differences may determine the rate of muscle deterioration, intervention response, and long-term prognosis. Therefore, future management strategies should shift from “single-disease diagnosis and treatment” toward “risk stratification and individualized intervention” (57, 58). To translate the principles of precision medicine into practical clinical applications, a phased management framework for DKD patients with sarcopenia was constructed by integrating recommendations from ADA 2026, KDIGO 2024, EWGSOP2, and AWGS2025, defining core objectives to guide clinical assessment and intervention in the absence of disease-specific guidelines(**Figure 9**, more details see **Supplementary Table 2**).

Building upon this framework, the study outlines an integrated three-stage management pathway. Stage one focuses on screening and assessment, using UACR and eGFR for kidney evaluation (21), alongside tools such as the SARC-F questionnaire, grip strength, and chair stand tests for rapid sarcopenia screening (22). Stage two addresses diagnosis and risk stratification. Following KDIGO 2024 and EWGSOP2 standards, patients are stratified by eGFR, albuminuria (20), and sarcopenia severity (muscle weakness, mass loss, and functional impairment) (22). Notably, In sarcopenic patients, reduced muscle mass may limit creatinine production, potentially biasing creatinine-based eGFR; thus, eGFRcr-cys or cystatin C–based eGFR is recommended for more accurate assessment (20). Stage three emphasizes interventions via a “cardio-renal-muscle” dual-protection strategy. Pharmacological treatment, per ADA 2026 and KDIGO 2024, includes SGLT2 inhibitors, nonsteroidal MRAs, ACEIs/ARBs, and GLP-1 receptor agonists (20, 21). Lifestyle modifications prioritize resistance training and a personalized nutritional regimen (0.8 g/kg/day for non-dialysis, 1.0–1.2 g/kg/day for dialysis), emphasizing high-quality protein within renal limits (20). The corresponding assessment tools and exploratory frameworks are presented in **Tables 4a**, **4b**, and **5**.

**Table 4a:** DKD combined with sarcopenia: clinical assessment indicators and tools based on current guidelines.

Assessment domain	Specific indicators/tools	Clinical application	Guideline source
Renal Function	eGFR, UACR, Serum Creatinine, Cystatin C	DKD screening, staging, and cardiorenal risk stratification	KDIGO 2024/ADA 2025
Metabolic Control	HbA1c < 7%, Blood Pressure < 130/80 mmHg	DKD glycemic and blood pressure targets	ADA 2025
Nutritional Status	7-Point SGA/MIS/MNA	Screening and comprehensive assessment of malnutrition risk in CKD patients	KDIGO 2024
Sarcopenia Screening	SARC-F questionnaire (≥4 points), SARC-CalF, Calf Circumference (Men <34 cm, Women <33 cm)	Preliminary identification of individuals at risk for sarcopenia	EWGSOP2/AWGS 2025
Muscle Strength Assessment①	Handgrip: Men <27 kg, Women <16 kg; Five Times Sit-to-Stand Test: >15 s	①: Probable Sarcopenia	EWGSOP2
Muscle Mass Assessment②	DXA measured ASM: Men <20 kg, Women <15 kg;	①+②: Confirmed Sarcopenia	EWGSOP2
	DXA measured ASMI: Men <7.0 kg/m², Women <5.5 kg/m²		
Physical Function	Gait Speed (≤0.8 m/s), Five Times Sit-to-Stand Test (>15 s), TUG (≥20 s), SPPB (≤8 points)	①+②+③: Severe Sarcopenia	EWGSOP2

ASM, appendicular skeletal muscle mass; ASMI, appendicular skeletal muscle mass index; AWGS, Asian Working Group for Sarcopenia; CKD, chronic kidney disease; DXA, dual-energy X-ray absorptiometry; eGFR, estimated glomerular filtration rate; EWGSOP2, European Working Group on Sarcopenia in Older People, 2nd Edition; HbA1c, hemoglobin A1c; KDIGO, Kidney Disease Improving Global Outcomes; MNA, Mini Nutritional Assessment; MIS, malnutrition-inflammation score; SGA, subjective global assessment; SPPB, Short Physical Performance Battery; TUG, Timed Up and Go test; UACR, urine albumin-to-creatinine ratio; SARC-F, Strength Assistance with walking Rise from a chair Climb stairs Falls questionnaire; SARC-CalF, SARC-F plus calf circumference.

This table lists clinical assessment indicators endorsed by KDIGO 2024, ADA 2025, EWGSOP2, and AWGS 2025 for evaluating renal function, metabolic control, nutritional status, and sarcopenia (screening, muscle strength, mass, and physical function) in patients with diabetic kidney disease and sarcopenia. All indicators are for assessment only, not therapeutic targets. Sarcopenia diagnosis follows the EWGSOP2 framework: low muscle strength (①) indicates probable sarcopenia; confirmed (①+②) requires low muscle mass; severe (①+②+③) adds poor physical performance. For nutritional status, KDIGO 2024 recommends composite tools (7−Point SGA, MIS, or MNA) over isolated laboratory markers. Thresholds should be applied with clinical judgment.

**Table 4b:** Candidate biomarkers and systems biology integrative research framework for DKD combined with sarcopenia.

Framework level	Biomarker combination	Candidate markers/variables	Biological or clinical rationale	Evidence status in this study	Research prospect
Extended Clinical Biomarkers	Tubular Injury	Urine KIM-1, NGAL (59, 60)	Reflects renal tubular injury and may precede a decline in eGFR.	Bibliometric studies focus on kidney dysfunction, dialysis, proteinuria, and DKD progression.	Specific evidence for DKD combined with sarcopenia is insufficient; prospective validation studies needed
	Inflammation & Nutritional Status	CRP, IL-6, TNF-α, Albumin, Prealbumin, Neutrophil-to-Lymphocyte Ratio (61–64)	Reflects chronic inflammation, catabolism, and nutritional deficiency; associated with CKD-related muscle wasting and sarcopenia.	Consistent with clinical evidence linking increased inflammation and catabolic pathways to CKD-related muscle loss.	Not explicitly recommended for routine testing in guidelines; requires validation to assess specificity for DKD combined with sarcopenia.
Metabolic & Endocrine Biomarkers	Insulin resistance and IGF signaling	HOMA-IR, IGF-1, Insulin/Glucose Ratio (65, 66)	Represents insulin resistance and impaired IGF signaling affecting kidney injury and muscle protein metabolism	Linked to PI3K-Akt, FoxO, MAPK, and AGE-RAGE signaling pathways	Requires validation of causal relationships and clinical predictive value in patients with DKD combined with sarcopenia.
Cellular/Molecular Damage Biomarkers	Oxidative stress and mitochondrial dysfunction	8-OHdG, MDA, SOD, GPx, Mitochondrial DNA Copy Number, or Circulating Mitochondrial Injury Markers (42, 43, 67, 68)	Oxidative stress and mitochondrial damage may lead to myocyte apoptosis, muscle atrophy, and impaired energy metabolism.	Proposed based on pathway-level interpretations; its validity as a diagnostic panel has not been verified in this study.	Not yet recommended by clinical guidelines for routine diagnosis and treatment; validation in multi-omics cohorts is needed to assess diagnostic performance and predictive value for intervention response.
Systems Biology and Integrative Models	Network-Derived Molecular Features	AKT1, STAT3, CTNNB1, INS, EGFR, TP53 and Related Pathway Activity Scores (49, 50)	Located within shared signaling networks related to inflammation, metabolism, apoptosis, and cellular stress.R, TP53, and Related Pathway Activity Scores	Derived from GeneCards, STRING PPI topology, and enrichment analysis; lacks specificity for expression directionality.	Experimental validation is required to confirm expression direction and functionality.
	Multi-Omics Combination	Transcriptomic, Proteomic, and Metabolomic Features (e.g., Mitochondria-Related Genes HTT, TTC19) (69)	Integrates multidimensional data to uncover molecular mechanisms of kidney-muscle cross-talk.	The original multi-omics data were not generated in this study; it is a hypothesis-generating integrative framework.	At an exploratory research stage; a dedicated DKD+SP cohort is needed for multi-omics analyses.
	Comprehensive Risk Assessment Model	eGFR/UACR + Muscle Indicators + Inflammatory Markers + Selected Molecular Readouts (46)	Develops a multi-level predictive model for early detection and personalized management.	Proposed based on integrative bibliometric and bioinformatics research findings, presenting a translational framework.	Conceptual framework; requires prospective cohort validation to assess calibration, discrimination, and clinical utility.

The biomarkers and integrative framework proposed in this table are strictly distinct from the content recommended by current clinical guidelines and fall within the scope of prospective research. Although the listed biomarkers have preliminary research evidence in single diseases (DKD or sarcopenia), their clinical value in DKD combined with sarcopenia has not been sufficiently validated. Currently, these biomarkers are not included in routine diagnostic and therapeutic recommendations by guidelines such as KDIGO, ADA, or EWGSOP2. This framework is constructed based on a hierarchical logic of “from clinically measurable indicators to molecular mechanisms and integrative models,” aiming to provide directional guidance for future precision medicine research. The basis for proposing these biomarkers primarily includes bibliometric analysis, bioinformatic prediction, or fundamental mechanism studies. These findings are more suitable for exploratory research or hypothesis generation, and their application in current clinical decision-making requires further validation.

**Table 5:** Proposed future research directions, staging model, and risk stratification algorithm for the diabetic kidney disease-sarcopenia axis.

Component	Proposed element	Suggested implementation or indicators	Expected value
Future research roadmap	Standardized epidemiology	Use unified diagnostic criteria for DKD and sarcopenia; conduct multicenter cohorts with longitudinal follow-up; report prevalence, incidence, and prognostic outcomes using comparable definitions.o (32, 70, 71)	Improve comparability across studies and clarify the true clinical burden of the DKD-sarcopenia phenotype.
Future research roadmap	Deeper phenotyping	Assess body composition, muscle mass, handgrip strength, gait speed/SPPB, renal stage, albuminuria, nutritional status, frailty, dialysis status, and treatment exposure (20, 72–74).	Identify heterogeneous phenotypes and distinguish early vulnerability from established functional decline.
Future research roadmap	Multi-omics integration	Integrate transcriptomics, proteomics, metabolomics, and, where available, single-cell or spatial omics with clinical phenotypes and renal-muscle outcomes.e (22, 75–77)	Move from descriptive association to mechanism-informed classification and biomarker discovery.
Future research roadmap	Mechanism validation	Prioritize experimental validation of inflammation and oxidative stress, insulin resistance/PI3K-Akt/FoxO signaling, AGE-RAGE/HIF-1 pathways, mitochondrial dysfunction, and muscle-kidney crosstalk (26, 28, 32).	Reduce overinterpretation of bibliometric or bioinformatic signals and identify biologically plausible intervention targets.
Future research roadmap	Biomarker panel development	Combine candidate genes and pathways with measurable biomarkers, including HbA1c, albumin, CRP, IL-6, TNF-alpha, eGFR, and UACR (46).	Develop practical, parsimonious biomarker signatures suitable for clinical risk assessment.
Future research roadmap	Clinical translation	Design prospective studies and trials testing exercise, nutrition, renal-protective management, and pharmacologic strategies within guideline-informed multidisciplinary care (26).	Translate mechanism-informed evidence into earlier detection, personalized management, and improved outcomes.
Proposed staging model	Stage 0: At-risk state	Aging, type 2 diabetes, obesity or malnutrition, reduced physical activity, chronic inflammation, or dialysis/uremic burden; no confirmed DKD-sarcopenia comorbidity.	Support primary prevention and baseline screening before overt renal-muscle deterioration.
Proposed staging model	Stage 1: Early renal-muscle vulnerability	Early albuminuria or eGFR decline and/or reduced muscle reserve without clear functional disability; possible abnormalities in nutrition or inflammatory markers.	Enable early lifestyle, nutritional, metabolic, and renal-protective intervention.
Proposed staging model	Stage 2: Established DKD-sarcopenia comorbidity	Coexistence of DKD with low muscle mass, reduced handgrip strength, slow gait speed, or low SPPB score.	Define the core clinical phenotype requiring coordinated nephrology, endocrinology, nutrition, and rehabilitation management.
Proposed staging model	Stage 3: Accelerated metabolic-renal-muscle deterioration	Progressive eGFR decline or worsening UACR together with functional decline, poor nutritional reserve, insulin resistance, inflammatory activation, or reduced glucose utilization.	Identify patients needing intensified monitoring and individualized treatment adjustment.
Proposed staging model	Stage 4: Adverse outcome stage	Frailty, falls, hospitalization, cardiovascular events, dialysis progression, or elevated mortality risk in the context of DKD-sarcopenia (71).	Guide high-risk follow-up, comprehensive geriatric-nephrology care, and outcome-focused interventions.
Risk stratification algorithm	Core risk domains	Include renal severity (eGFR, UACR), muscle status (ASM/SMI, handgrip strength, gait speed/SPPB), metabolic status (HbA1c, insulin resistance), nutrition/inflammation (albumin, CRP, IL-6, TNF-alpha), and clinical vulnerability (age, comorbidity, physical inactivity, dialysis/uremic burden).	Provide a structured basis for clinical scoring and individualized risk classification.
Risk stratification algorithm	Suggested risk categories	Low risk: risk factors without functional decline; intermediate risk: DKD or sarcopenia with one additional abnormal domain; high risk: confirmed DKD-sarcopenia or multiple abnormal domains; very high risk: rapid renal decline, severe albuminuria, frailty, falls, hospitalization, or major cardiovascular risk.	Link risk level to screening frequency, intervention intensity, and referral priority.
Risk stratification algorithm	Model development and validation	Derive candidate scores in multicenter cohorts; compare additive clinical scores with machine-learning models; evaluate discrimination, calibration, decision-curve utility, and external validity; test clinical effectiveness prospectively (77, 78).	Ensure that the proposed algorithm becomes a validated, clinically usable tool rather than a conceptual framework only.

This proposed roadmap, staging model, and risk stratification algorithm is a conceptual and hypothesis-generating framework derived from bibliometric patterns, exploratory bioinformatic signals, and current clinical assessment concepts. It should not be interpreted as a validated diagnostic criterion, clinical staging system, prognostic model, or treatment algorithm. The proposed stages are intended to organize future research and clinical translation across renal severity, muscle phenotype, metabolic disturbance, nutritional and inflammatory burden, functional decline, and adverse outcomes. Prospective multicenter cohorts and interventional studies are required to validate the proposed risk domains, refine stage definitions, evaluate predictive performance, and determine whether stage-guided management improves renal, metabolic, functional, and patient-centered outcomes. DKD, diabetic kidney disease; ASM, appendicular skeletal muscle mass; SMI, skeletal muscle index; SPPB, Short Physical Performance Battery; UACR, urinary albumin-to-creatinine ratio; eGFR, estimated glomerular filtration rate; CRP, C-reactive protein; IL-6, interleukin-6; TNF-α, tumor necrosis factor-α.

### 4.9 Future directions: from standardization to multidisciplinary translation

**Figure 10:**
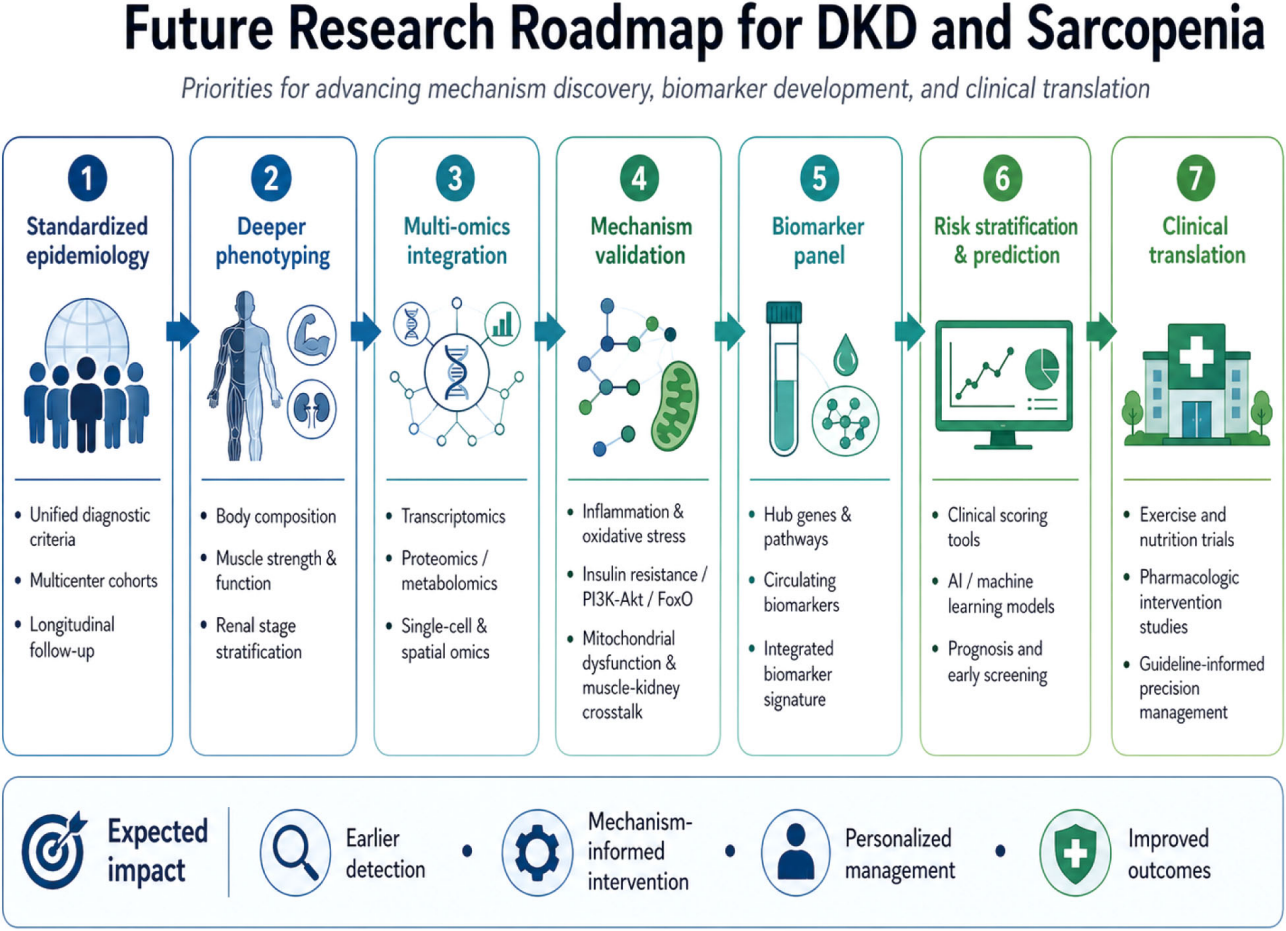
Future research roadmap for diabetic kidney disease and sarcopenia.

**Figure 11:**
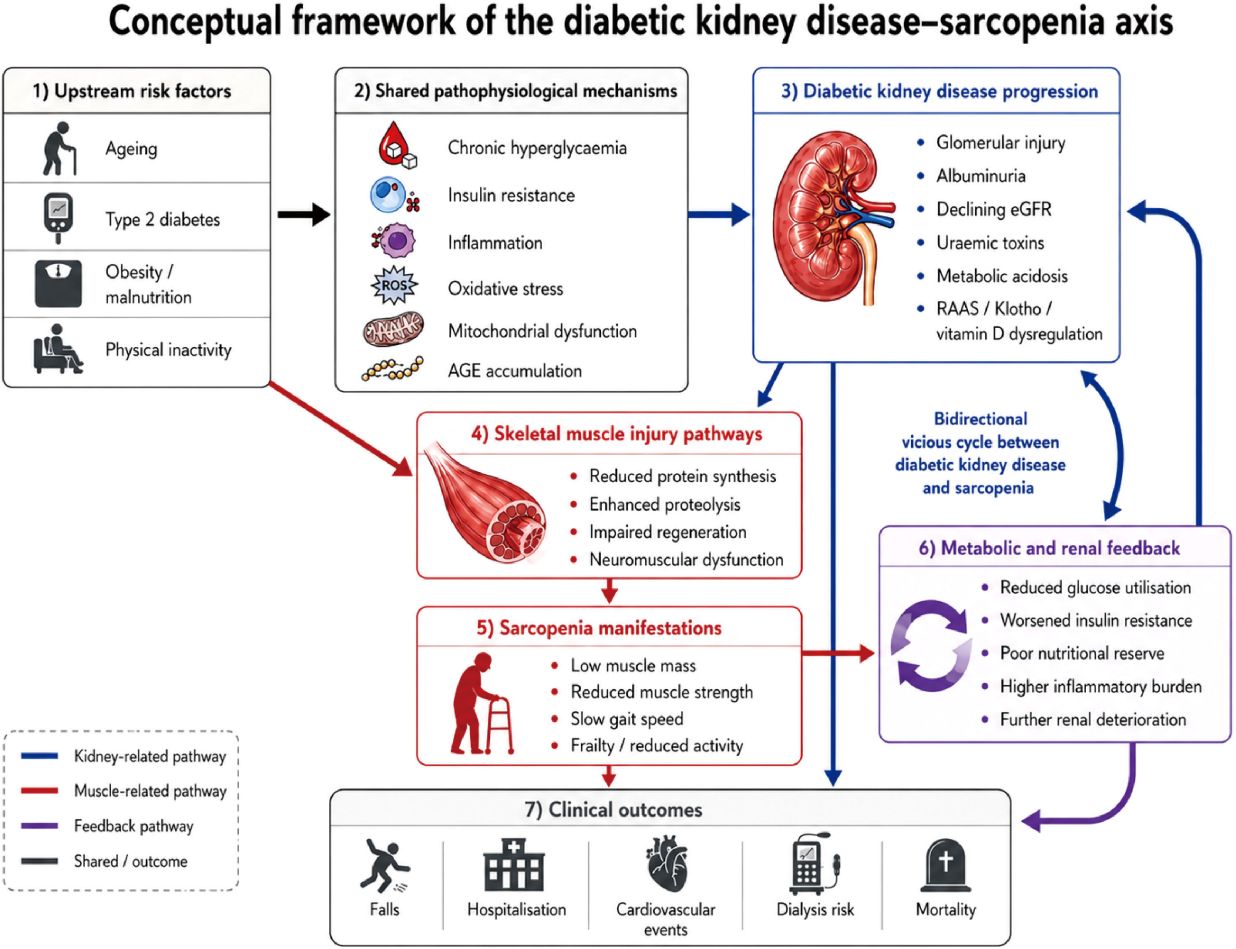
Proposed conceptual model of the diabetic kidney disease–sarcopenia axis.

Based on the results of this study and existing evidence, future research on the intersection between DKD and sarcopenia may be further advanced in the following directions (**Figure 10**). First, research definitions and measurement standards should be unified. Inconsistencies across studies in the definitions of DKD, chronic kidney disease stage, sarcopenia, sarcopenic obesity, and protein-energy wasting represent important obstacles to current evidence integration. Future studies should prioritize the use of international consensus criteria and clearly report measurement methods for muscle strength, muscle mass, and physical function. Second, longitudinal cohorts and causal inference studies should be strengthened. Many current studies are cross-sectional, making it difficult to determine the temporal sequence between DKD progression and muscle deterioration. Long-term follow-up cohorts are needed to dynamically observe changes in blood glucose, renal function, inflammation, nutritional status, and muscle phenotypes, thereby clarifying which factors can predict the occurrence, progression, and adverse outcomes of sarcopenia. Third, mechanistic research should be better integrated with clinical studies. Omics and molecular mechanism studies can provide potential targets, but these must be validated through independent cohorts, experimental models, and clinical phenotypes. In the future, transcriptomics, proteomics, metabolomics, gut microbiota, and clinical functional indicators may be integrated to construct a multidimensional mechanistic network for DKD-associated sarcopenia. It may be worthwhile to connect these mechanistic findings with intervention measures endorsed by current guidelines, such as exploring the impact of SGLT2 inhibitors on muscle metabolomics. Fourth, interventional trials targeting the intersecting population should be conducted. Future clinical trials should not evaluate only blood glucose, proteinuria, or eGFR, but should also include endpoints such as handgrip strength, gait speed, muscle mass, physical function, quality of life, and frailty status. In particular, the effects of SGLT2 inhibitors, GLP-1 receptor agonists, nutritional interventions, resistance training, and comprehensive rehabilitation programs on muscle health should be evaluated. Finally, public health translation and clinical application should be strengthened. DKD complicated by sarcopenia is not only a nephrology or endocrinology issue, but also involves geriatrics, nutrition, rehabilitation medicine, and public health management. In the future, multidisciplinary collaborative pathways should be established, sarcopenia screening should be incorporated into the routine management of patients with DKD, and feasible and scalable intervention strategies should be developed according to risk stratification. Overall, research on the intersection between DKD and sarcopenia remains in a phase of rapid development. Through standardized assessment, mechanistic validation, multi-omics integration, and precision intervention, this field is expected to progress from descriptive research toward mechanistic elucidation and clinical translation. **Figure 11** offers more details.

### 4.10 Limitations

This study has several limitations.

This study has several limitations. First, although we searched three databases, including WoSCC, Scopus, and PubMed, to expand literature coverage and reduce single-database bias, differences still exist among databases in coverage scope, indexing rules, citation statistics, and field structures. Therefore, cross-database integration cannot completely eliminate database coverage bias. In particular, PubMed is not fully consistent with WoSCC and Scopus in terms of citation fields and reference structures. Based on this, the present study handled general bibliometric analysis and citation performance analysis separately. Except for citation analysis, annual publication trends, country/region distribution, author, institution, journal, and keyword analyses were mainly based on the merged three-database dataset, whereas citation performance analysis was mainly descriptively summarized based on available and relatively consistent citation information.

Second, this study included only English-language publications, which may have caused certain language and regional biases. Some studies on DKD and sarcopenia from non-English-speaking countries or regions may not have been fully included, thereby affecting the interpretation of country/region distribution, institutional contributions, and collaboration network results. Future studies may further include multilingual and regional databases to obtain a more comprehensive global research map.

Third, although countries/regions, institutions, journals, and keywords were standardized in this study, cross-database bibliographic information may still contain inconsistencies in author name abbreviations, institutional name variants, keyword expressions, and address field parsing errors. In particular, in the absence of unique author identifiers such as ORCID, a small number of errors may still exist in the author collaboration network, such as the incorrect merging of authors with the same name or incomplete merging of the same author. Therefore, author and institutional collaboration results are more appropriately interpreted at the macro level of collaboration structure, rather than being overinterpreted for individual nodes.

Fourth, bibliometric analysis essentially reflects research activity, academic attention, and changes in knowledge structure, but cannot directly prove disease mechanisms or causal relationships. Keyword co-occurrence, thematic clustering, and burst term analysis can suggest research hotspots and potential frontiers, but their results are influenced by search strategy, database coverage, keyword standardization, and algorithm parameter settings. Therefore, in interpreting keyword and thematic evolution results, this study mainly treated them as descriptive evidence of research trends and knowledge structures, rather than direct evidence of biological mechanisms.

Fifth, indicators such as publication volume, country/region contribution, institutional output, author productivity, journal distribution, keyword frequency, and citation ranking in this study were mainly descriptive counts based on database records, rather than inferential statistical results based on sampled data. Therefore, confidence intervals or uncertainty intervals were not calculated for all bibliometric indicators. Annual publication trend fitting was mainly used to assist in displaying temporal changes in research output, rather than to predict future publication volume or perform causal inference. Future studies may further introduce bootstrap stability analysis, parameter sensitivity analysis, and leave-one-database-out sensitivity analysis to enhance result robustness.

Sixth, citation indicators have inherent limitations. Global citation counts and local citation counts are easily affected by publication time, journal impact, disciplinary dissemination scope, and citation lag effects. Earlier publications usually have longer time for citation accumulation, while high-quality studies published in recent years may not yet have received sufficient citations. Therefore, highly cited status does not necessarily represent the highest level of evidence or the strongest clinical value. To reduce interpretation bias caused by reliance on a single citation indicator, this study combined annual trends, keyword evolution, and thematic structure to comprehensively judge changes in research hotspots and frontiers.

Seventh, here are exploratory limitations in bioinformatic analyses. In this study, bioinformatic analyses were purely exploratory and intended for hypothesis generation, using disease-related genes mined exclusively from the GeneCards database with relevance score thresholds of DKD ≥ 39.4 and sarcopenia ≥ 63.0. It is important to note that GeneCards, being an integrative database, collects information from diverse heterogeneous sources. Consequently, potential curation biases and selective biases may exist in gene-disease association scores, data update frequency, and overall coverage.Moreover, the analyses capture only associations and network topology at the database level, without indicating expression direction, tissue specificity, or causal relationships. Consequently, shared genes and pathway enrichment results should be regarded as indicative of associations at the database level and as tools for generating research hypotheses, not as direct proof of disease mechanisms.Future studies aiming for validation should combine clinical specimens, multi-omics datasets, and functional experiments to rigorously confirm these findings.

## 5 Conclusion

This study integrates multi-database bibliometric analysis along with exploratory and hypothesis-driven bioinformatics research to systematically present the trends in research outputs, collaboration patterns, thematic hotspots, and potential molecular links in the intersecting field of DKD and sarcopenia. The analysis reveals a growing scholarly focus on the DKD-sarcopenia interface, with research topics shifting from initial epidemiological observations and clinical association studies to a more nuanced exploration of dysregulated nutritional and inflammatory states (NIS), decline in physical function, prognostic implications, exercise and dietary interventions, and shared molecular signatures.

The findings suggest that the DKD–sarcopenia axis may extend beyond a simple comorbid relationship. Instead, it likely reflects shared pathophysiological mechanisms involving metabolic dysregulation, chronic inflammation, and mitochondrial dysfunction. Based on trends in thematic evolution and network analysis, this study proposes a conceptual framework for the DKD–sarcopenia axis, alongside a clinical translation framework, a research roadmap, and a staging framework. These tools aim to bridge knowledge gaps in the field, transitioning from identifying research hotspots to elucidating mechanisms and informing translational and clinical applications.

Nevertheless, it is essential to emphasize that the DKD–sarcopenia axis, candidate gene pathways, and staging model remains in the hypothesis-generating phase. The proposed clinical translation framework integrates stage-specific management practices from established clinical guidelines for each disease, while the alignment of management objectives across the DKD-sarcopenia continuum represents a novel constructed framework. Robust validation through multicenter prospective cohort studies, standardized phenotyping, functional assays, and risk modeling incorporating clinical outcomes will be critical for their clinical translation.

From a clinical perspective, strategies to manage DKD should expand beyond improving kidney function and addressing proteinuria. A more holistic approach is vital, incorporating assessments of muscle mass, strength, physical performance, nutritional status, and inflammatory burden. The successful management of DKD with concurrent sarcopenia will require interdisciplinary collaboration among nephrology, endocrinology, geriatrics, and rehabilitation medicine. Such efforts have the potential to advance the field from conceptual frameworks to mechanistic perspectives, enabling early detection, risk stratification, and tailored interventions.
