# Supplementary material for: Research trends and potential molecular intersections between diabetic kidney disease and sarcopenia: a 21-year bibliometric and bioinformatics analysis
Source: Front Endocrinol (Lausanne). 2026 Jun 9;17:1798210. doi: 10.3389/fendo.2026.1798210 (PMC13286762; doi:10.3389/fendo.2026.1798210)
Supplement: Supplementary file 1 [file Table1.docx]

**Supplementary Material**

**Supplementary Table S1. Search strategies**

| **Database** | **Search strategy** | **Limits / notes** |
| --- | --- | --- |
| **Web of Science Core Collection** | TS=(  (  "Diabetic Nephropath*"  OR "Diabetic Kidney Disease"  OR "Diabetic Renal Disease"  OR "Diabetes-Related Kidney Disease"  OR "Diabetes-Associated Kidney Disease"  OR "Chronic Diabetic Nephropathy"  OR "Kidney Failure in Diabetes"  OR "Renal Dysfunction in Diabetes"  OR "Diabetic Kidney Dysfunction"  OR "Diabetic Renal Impairment"  OR "Nephropathy in Diabetes"  OR ((diabet* NEAR/3 (kidney OR renal)) NEAR/3 (disease* OR dysfunction* OR impair* OR failure* OR nephropath*))  )  AND  (  sarcopeni*  OR cachexi*  OR "muscle wast*"  OR "muscle atroph*"  OR "muscle mass loss"  OR "muscle deplet*"  OR "skeletal muscle deplet*"  OR "skeletal muscle wast*"  OR "skeletal muscle dysfunction"  OR "muscle weakness"  OR "muscle strength"  OR "muscle loss"  OR "muscle degeneration"  ) ) | Topic search (TS). Final records were limited to English-language articles and reviews and excluded records published after December 31, 2025 during screening/export. |
| **PubMed** | (  "Diabetic Nephropathies"[Mesh]  OR "diabetic nephropath*"[Title/Abstract]  OR "diabetic kidney disease"[Title/Abstract]  OR "diabetic renal disease"[Title/Abstract]  OR "diabetes-related kidney disease"[Title/Abstract]  OR "diabetes-associated kidney disease"[Title/Abstract]  OR "chronic diabetic nephropathy"[Title/Abstract]  OR "kidney failure in diabetes"[Title/Abstract]  OR "renal dysfunction in diabetes"[Title/Abstract]  OR "diabetic kidney dysfunction"[Title/Abstract]  OR "diabetic renal impairment"[Title/Abstract]  OR "nephropathy in diabetes"[Title/Abstract]  OR (  diabet*[Title/Abstract]  AND (kidney[Title/Abstract] OR renal[Title/Abstract])  AND (disease*[Title/Abstract] OR dysfunction*[Title/Abstract] OR impair*[Title/Abstract] OR failure*[Title/Abstract] OR nephropath*[Title/Abstract])  ) ) AND (  "Sarcopenia"[Mesh]  OR "Cachexia"[Mesh]  OR sarcopeni*[Title/Abstract]  OR cachexi*[Title/Abstract]  OR "muscle wast*"[Title/Abstract]  OR "muscle atroph*"[Title/Abstract]  OR "muscle mass loss"[Title/Abstract]  OR "muscle deplet*"[Title/Abstract]  OR "skeletal muscle deplet*"[Title/Abstract]  OR "skeletal muscle wast*"[Title/Abstract]  OR "skeletal muscle dysfunction"[Title/Abstract]  OR "muscle weakness"[Title/Abstract]  OR "muscle strength"[Title/Abstract]  OR "muscle loss"[Title/Abstract]  OR "muscle degeneration"[Title/Abstract] ) AND English[Language] | Fields: MeSH Terms and Title/Abstract. Language limited to English. Final records were limited to articles and reviews and excluded records published after December 31, 2025 during screening/export. |
| **Scopus** | TITLE-ABS-KEY(  (  "diabetic nephropath*"  OR "diabetic kidney disease"  OR "diabetic renal disease"  OR "diabetes-related kidney disease"  OR "diabetes-associated kidney disease"  OR "chronic diabetic nephropathy"  OR "kidney failure in diabetes"  OR "renal dysfunction in diabetes"  OR "diabetic kidney dysfunction"  OR "diabetic renal impairment"  OR "nephropathy in diabetes"  OR ((diabet* W/3 (kidney OR renal)) W/3 (disease* OR dysfunction* OR impair* OR failure* OR nephropath*))  )  AND  (  sarcopeni*  OR cachexi*  OR "muscle wast*"  OR "muscle atroph*"  OR "muscle mass loss"  OR "muscle deplet*"  OR "skeletal muscle deplet*"  OR "skeletal muscle wast*"  OR "skeletal muscle dysfunction"  OR "muscle weakness"  OR "muscle strength"  OR "muscle loss"  OR "muscle degeneration"  ) ) AND (LIMIT-TO(DOCTYPE, "ar") OR LIMIT-TO(DOCTYPE, "re")) AND LIMIT-TO(LANGUAGE, "English") | Fields: Title, Abstract, and Keywords. Document types: article (ar) and review (re). Language: English. Records published after December 31, 2025 were excluded during screening/export. |

Abbreviations: MeSH, Medical Subject Headings; TS, topic search; TITLE-ABS-KEY, title, abstract, and keywords.
