# Supplementary material for: Research trends and potential molecular intersections between diabetic kidney disease and sarcopenia: a 21-year bibliometric and bioinformatics analysis
Source: Front Endocrinol (Lausanne). 2026 Jun 9;17:1798210. doi: 10.3389/fendo.2026.1798210 (PMC13286762; doi:10.3389/fendo.2026.1798210)
Supplement: Supplementary file 2 [file Table2.docx]

Supplementary Material

**Supplementary Table 2. Translational Clinical Framework for the Management of Diabetic Kidney Disease (DKD) and Sarcopenia: From Screening to Intervention**

| **Stage** | **Core Content** | **Clinical Practice** | | | **Evidence Level / Notes & Guidelines** |
| --- | --- | --- | --- | --- | --- |
| Screening and Assessment | Objective: To identify the risk of DKD and sarcopenia early in high-risk populations. | Baseline Assessment of DKD | Urine albumin/creatinine ratio (UACR): Measured at the time of diagnosis for T2D patients; start testing in T1D patients with disease duration ≥5 years; all diabetic patients should be rechecked annually. | | Evidence level: B [ADA 2026] Note: Regardless of confirmed DKD, annual reassessment is recommended for monitoring. |
|  |  |  | eGFR: Estimate renal function using combined serum creatinine (CKD-EPI equation, without racial adjustment). | | [ADA 2026] |
|  |  | Preliminary Screening and Assessment of Sarcopenia | SARC-F questionnaire: Score ≥4 indicates high risk. | | Suitable for community and clinical use; negative results indicate no sarcopenia; predictive sensitivity for low muscle strength is low to moderate, but specificity is very high; mainly detects severe cases; cost-effective and convenient. [EWGSOP2] |
|  |  |  | Clinical suspicion: Focus on patient-reported symptoms or signs such as falls, feeling weak, slow gait, difficulty rising from chair. | | [EWGSOP2] |
|  |  |  | Handgrip strength test | | Normal: no sarcopenia, reassess later [EWGSOP2] |
|  |  |  | Chair stand test | |  |
|  |  |  | Ishii screening test | | Note: a more formal case finding instrument [EWGSOP2] |
| Diagnosis and Risk Stratification | Objective: Stratify DKD risk based on KDIGO 2024 and EWGSOP2 standards; clearly identify DKD and sarcopenia, and perform risk assessment and stratification based on eGFR, albuminuria, and muscle function indices. | DKD Comprehensive Risk Stratification | Risk stratification: Based on eGFR and albuminuria; reduced muscle mass may affect creatinine levels, consider using eGFRcys to obtain more accurate kidney function assessment. | | [KDIGO 2024 / ADA 2026] |
|  |  |  | Target value: Use a ≥30% reduction in UACR as a key intermediate marker for slowing DKD progression. | | Note: Mainly for patients with UACR ≥300 mg/g [ADA 2026] |
|  |  | Sarcopenia Diagnosis and Severity Classification | Low muscle strength：Probable Sarcopenia | Handgrip strength: Men <27 kg, Women <16 kg abnormal | Note: Muscle strength measurement metrics[EWGSOP2] |
|  |  |  |  | Chair stand test: 5 repetitions >15 s |  |
|  |  |  | Low muscle strength+ low muscle quantity or quality:Confirmed Sarcopenia | ASM: Men <20 kg / Women <15 kg | [EWGSOP2]Note: Low muscle mass measured by DXA |
|  |  |  |  | ASM/Height²: Men <7 kg/m², Women <5.5 kg/m² |  |
|  |  |  | Low muscle strength +Low muscle quantity or quality +Low physical performance：Severe Sarcopenia | Gait speed (4-meter walk test) ≤0.8 m/s | Note:Physical function metrics[EWGSOP2] |
|  |  |  |  | SPPB points ≤ 8 |  |
|  |  |  |  | TUG≥ 20 s |  |
|  |  |  |  | 400-meter walk test: not completed or ≥6 minutes |  |
| Intervention | Objective: Implement a multidisciplinary management strategy with renal protection as the core and muscle health as an adjunct, through pharmacotherapy, nutrition, and exercise interventions, to slow disease progression and improve patient outcomes. | Pharmacological Intervention | SGLT2 inhibitors: Recommended for T2D patients with CKD and eGFR ≥20 mL/min/1.73m² to reduce kidney disease progression and cardiovascular risk | | Evidence level A [ADA 2026, KDIGO 2024] |
|  |  |  | ns-MRA: For T2D patients with eGFR >25 mL/min/1.73m², normal serum potassium, and UACR >30 mg/g, reduces risk of cardiorenal complications | | Evidence level A [ADA 2026, KDIGO 2024] |
|  |  |  | ACEI/ARB: Recommended for patients with hypertension and albuminuria; severe albuminuria and/or eGFR<60 mL/min strongly recommended; titrate to maximum tolerated dose | | Evidence level A [ADA 2026] |
|  |  |  | GLP-1 receptor agonists: Recommended for T2D with CKD using agents with proven cardiorenal benefits (e.g., semaglutide) to reduce kidney disease progression and cardiovascular risk | | Evidence level A [ADA 2026]; caution if eGFR<15 or dialysis |
|  |  |  | Sarcopenia-targeted pharmacotherapy: Currently, no approved drugs are available. Investigational drugs, such as losartan (preclinical studies), show potential in reducing muscle fibrosis. | | Clinical pre-research stage [AWGS 2025][AWGS 2025] |
|  |  | Lifestyle Intervention | Exercise Intervention | Resistance training: Increases muscle mass, lower limb strength, and physical function | [AWGS 2025] |
|  |  |  |  | Combined exercise: Resistance + aerobic or balance training to improve gait speed and quality of life | [AWGS 2025] |
|  |  |  | Nutrition | DKD priority (non-dialysis): Protein intake limit 0.8 g/kg/day; suitable for CKD G3+ patients | Evidence level A [ADA 2026] |
|  |  |  |  |  | Evidence level A [ADA 2026] |
|  |  |  |  | DKD priority (dialysis): Protein intake 1.0–1.2 g/kg/day, supplement protein for sarcopenia as appropriate | Evidence level B [ADA 2026] |
|  |  |  |  | Sarcopenia considerations: Appropriate protein supplementation | [AWGS 2025] |
|  |  |  |  | Integrated strategy: For diabetic patients with sarcopenia, under nephrologist guidance, ensure optimal protein intake (0.8–1.2 g/kg/day within CKD limits), combined with resistance exercise to improve protein utilization. | Note: Individualized adjustment required to balance kidney protection and muscle synthesis needs. |

Abbreviations:

ACEI, angiotensin-converting enzyme inhibitor; ARB, angiotensin II receptor blocker; ASM, appendicular skeletal muscle mass; BIA, bioelectrical impedance analysis; CKD, chronic kidney disease; DKD, diabetic kidney disease; DXA, dual-energy X-ray absorptiometry; eGFR, estimated glomerular filtration rate; GLP-1 RA, glucagon-like peptide-1 receptor agonist; MRA, mineralocorticoid receptor antagonist; ns-MRA, non-steroidal mineralocorticoid receptor antagonist; SPPB, Short Physical Performance Battery; T1D, type 1 diabetes; T2D, type 2 diabetes; TUG, Timed Up and Go test; UACR, urine albumin-to-creatinine ratio; KDIGO, Kidney Disease: Improving Global Outcomes;EWGSOP2, European Working Group on Sarcopenia in Older People 2; AWGS, Asian Working Group for Sarcopenia.

Notes:

• Evidence levels are graded according to the American Diabetes Association (ADA) Standards of Care (2026) and KDIGO (2024) Clinical Practice Guidelines, where 'A' indicates high-quality evidence from well-conducted randomized controlled trials and 'B' indicates moderate-quality evidence from observational studies or randomized trials with limitations.

• Clinical cutoffs for sarcopenia diagnosis follow the European Working Group on Sarcopenia in Older People (EWGSOP2) criteria.

•Recommendations for sarcopenia management (exercise and nutrition) are based on the Asian Working Group for Sarcopenia (AWGS) 2025 consensus update, which emphasizes a shift from sarcopenia diagnosis to lifelong muscle health promotion across the lifespan.

• The integrated nutritional strategy aims to balance renal protection with muscle synthesis requirements; protein intake should be individualized based on kidney function stages and nutritional status.

• This framework integrates stage-specific management practices derived from established clinical guidelines for each disease, while the alignment of management objectives across the DKD–sarcopenia continuum represents a novel constructed model.
